# Understanding self-assembly across scales in BODIPY derivatives: from supramolecular aggregation in solution to optical-waveguiding crystalline materials

**DOI:** 10.1039/d5sc07776a

**Published:** 2025-11-28

**Authors:** Marina González-Sánchez, Ana M. Garcia, Jorge S. Valera, Ana M. Rodríguez, Juan Cabanillas-González, Pilar Prieto, David González-Rodríguez

**Affiliations:** a Nanostructured Molecular Systems and Materials, Organic Chemistry Department, Universidad Autónoma de Madrid 28049 Madrid Spain david.gonzalez.rodriguez@uam.es; b Facultad de Ciencias y Tecnologías Químicas, Instituto Regional de Investigación Científica Aplicada (IRICA), Universidad de Castilla-La Mancha Ciudad Real 13071 Spain mariapilar.prieto@uclm.es; c Madrid Institute for Advanced Studies, IMDEA Nanociencia, Ciudad Universitaria de Cantoblanco Calle Faraday 9 28049 Madrid Spain; d Institute for Advanced Research in Chemical Sciences (IAdChem), Universidad Autónoma de Madrid 28049 Madrid Spain

## Abstract

Molecular self-assembly constitutes a useful tool to construct discrete and functional nanostructures with well-defined properties. Nevertheless, understanding supramolecular order in solution and its transmission to the solid state for the same scaffold remains challenging and the impact of this transition in the properties of the resulting supramolecular architectures is still underexplored, thus limiting the application of self-assembly in fields like organic electronics. Herein we explore the supramolecular aggregation process of a chiral BODIPY derivative in solution, its features in solid state, and its potential application as optical waveguide. In solution, this derivative follows a time-dependent self-assembly process, ultimately leading to aggregates of increasing dimensionality with propensity to precipitate, from which a certain degree of crystallinity can be inferred. The subsequent formation of single crystals confirms the preservation of key supramolecular features from solution to the solid state. Notably, the one-centimeter-long crystals obtained present active optical waveguiding behavior in the NIR with a remarkable optical loss coefficient, the first optical loss reported for BODIPY crystals. These results highlight the value of studying self-assembly across different scales and demonstrate the potential of BODIPY-based systems for the development of optical waveguides with remarkable performance.

## Abstract 2

We report the self-assembly of a chiral BODIPY derivative from monomers to complex solution aggregates and crystalline waveguiding materials, highlighting the value of a multiscale understanding for functional π-conjugated systems.

## Graphical abstract

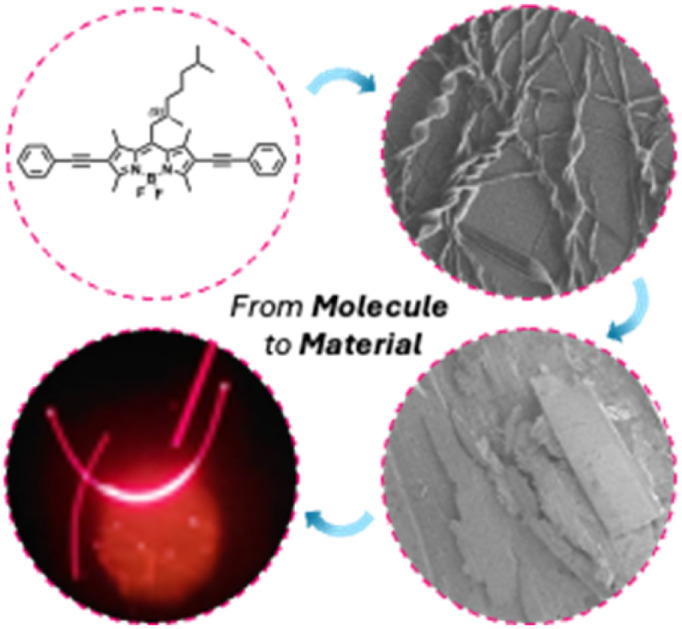

## Introduction

Molecular self-assembly allows the formation of well-defined architectures thanks to the information encoded in the different functional groups within the molecular structure.^1,2^ This process leads to the creation of supramolecular scaffolds with promising applications in electronics, sensing and nmcatalysis, to cite a few.^3,4^ Supramolecular polymers represent a particular class of 1D, self-assembled structures, formed through the establishment of non-covalent interactions between identical monomeric units.^5^ This field has been traditionally focused on the generation of dynamic supramolecular entities in solution, where significant progress has been made in understanding the relationship between molecular design and aggregate properties as well as controlling the kinetic and thermodynamic factors that drive their formation.^6–8^ However, despite these advancements, the translation of 1D supramolecular order in solution to the 3D order both in solution and in the solid state with discrete self-assembling systems remains largely unexplored, thus hampering the application of these scaffolds in organic electronics.^9,10^

Recent attempts to fill this gap have explored the nonclassical crystal growth of supramolecular fibers in aqueous medium.^11,12^ Nevertheless, the control over the different hierarchy levels of self-assembly in solution is still limited,^13,14^ and the resulting supramolecular fibers often aggregate into disordered solid structures or into gels at higher concentrations, states which cannot be considered (fully) crystalline.^15^ This difficulty stems from the different molecular designs required for achieving self-assembly in solution when compared to those which are prerequisite to achieve crystalline structures, and examples of molecules capable of both phenomena as well as the differences between both processes are scarce.^16^ Thus, comprehending how the features of a self-assembled structure transition from solution to the solid and crystalline states and its impact in the properties still remains a major challenge.

**Fig. 1:**
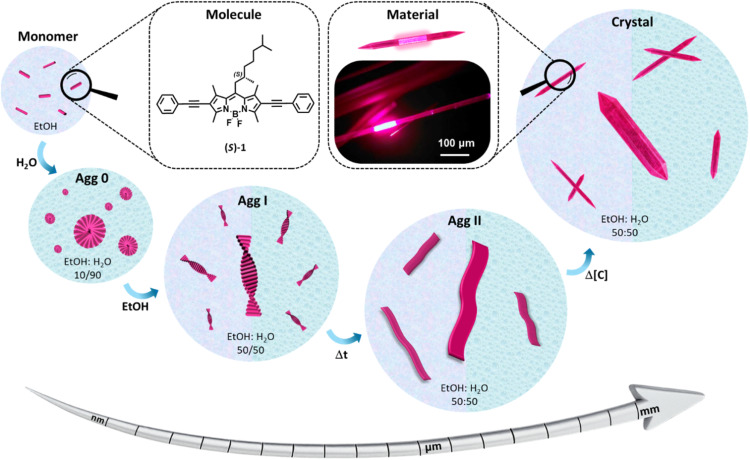
Chemical structure of the BODIPY derivative (*S*)-1 and schematic representation of the different self-assembled architectures in solution and solid state, obtained by varying the H_2_O/EtOH content, time or concentration, ultimately leading to crystalline materials with NIR optical waveguiding function.

A deeper understanding of these processes could be particularly valuable in optical waveguiding, where light is confined and transmitted over short-to-long distances within crystalline organic materials. Given the unique optoelectronic properties that they display, π-conjugated scaffolds are especially relevant for the field.^17–20^ In this context, derivatives of 4-difluoro-4-bora-3a,4a-diaza-*s*-indacene (BODIPY; Fig. 1) have received significant attention. Their excellent properties, comprising high thermal stability, photochemical stability, high molar absorption coefficients (usually *e* > 80 000 M^−1^ cm^−1^) and high fluorescence quantum yields (commonly *Φ* > 0.50), make them promising candidates for optical and electronic applications.^21–23^ Moreover, their unique molecular features permit to tune the formation of intermolecular interactions that in turn can lead to crystalline assemblies, and their use as optical waveguides has been already demonstrated.^24,25^ Although some optical waveguides have been reported, there are few examples of waveguides based on organic compounds that emit in the near-infrared (NIR) range (700–2500 nm). These materials have been used in medical treatments, as this type of light causes the least damage to biological tissues,^25^ as well as in 5G technologies.^26^ In this regard, the chemical versatility of the BODIPY core allows the development of compounds that emit in this region.^27^ Thus, establishing a direct link between the supramolecular assemblies of BODIPY derivatives in solution and those in the solid state, along with its optoelectronic and optical waveguiding behavior, could help to bridge the properties from the microscopic to the macroscopic scale, which may support the rational design of new materials for optoelectronic devices.

Herein we investigate the self-assembly behavior of the chiral BODIPY derivative (*S*)-1 in solution and also its behaviour in the solid state, ultimately evaluating its optical waveguiding performance (Fig. 1). The self-assembly process in solution, studied in EtOH/H_2_O mixtures, reveals the formation of distinct supramolecular architectures that strongly depend on both the preparation method and the H_2_O content. These variations lead to aggregates with different chiroptical features (ranging from small nuclei to helical H-type aggregates of increasing dimensionality) and diverse sizes and morphologies (from small globular entities to well-defined fibrillar structures of distinct widths), which eventually evolve to lamellar architecture with a certain degree of crystalline order and hence a tendency to precipitate. This prompted us to obtain single crystals at higher concentrations. The XRD patterns recorded in various solvents allowed us to establish direct correlations between the aggregation features observed in solution and those found in the solid state. Remarkably, we obtained one-centimeter-long crystals, which displayed optical waveguiding behavior with emission approaching the NIR range, and an optical loss coefficient of 0.016 dB µm^−1^. To our knowledge, among the few reported works on BODIPY waveguides described in the literature, this is the first optical loss value described for a crystalline BODIPY derivative. Thus, this molecular design provides a unique opportunity to explore self-assembly in solution, crystal formation and optical waveguiding properties, contributing to the broader understanding of self-assembly mechanisms across different states of matter.

## Results and discussion

### Synthesis and self-assembly in solution

The synthesis of the BODIPY derivative (*S*)-1 has been achieved in a multistep approach by partially reproducing previously reported synthetic routes^28–30^ (see Scheme S0 and Fig. S0A–S0F in the SI for further synthetic details and characterization). We initially assessed the spectroscopic features of (*S*)-1 in solution in different organic solvents by means of UV-Vis spectroscopy (Fig. S1a). In polar solvents like THF, EtOH and CH_3_CN and concentrations of 1.0 × 10^−5^ M, (*S*)-1 possesses two main absorption bands centered at 400 nm and 550 nm, corresponding to S_0_–S_2_ and to S_0_–S_1_ transitions of the BODIPY core, respectively.^21^ Surprisingly, these signatures remain unchanged even in apolar solvents like heptane, denoting the presence of the monomeric state of (*S*)-1 in these conditions.^31^ Fluorescence spectroscopy experiments (Fig. S1b) in the aforementioned conditions confirm the existence of (*S*)-1 in the monomeric state, yielding a strong emission band centered at 600 nm (*λ*_exc_ = 550 nm) and a high fluorescence quantum yield (*ϕ* = 0.51). Given the hydrophobicity of (*S*)-1 and in order to induce the self-assembly process, we explored the use of H_2_O as a cosolvent in the EtOH solutions. Increasing the water content to EtOH/H_2_O 70/30 leads to an initial decrease in both absorption and emission intensities, which is more pronounced in the EtOH/H_2_O 10/90 mixture (Fig. S1a and b). Under these conditions, the UV-Vis absorption band at 550 nm undergoes notable broadening and a slight bathochromic shift, splitting into two distinct bands with maxima at 540 nm and 575 nm. These spectral changes are accompanied by a substantial fluorescence quenching and a redshift of the main emission band to 640 nm, which is also visually evident by the changes in color exhibited in the prepared solutions.

We also profited from the chiral center in (*S*)-1 to gain further insight into the self-assembly mode in the EtOH/H_2_O 10/90 mixture by CD spectroscopy (Fig. S1c). In contrast to the negligible signal observed in the rest of solvents, the spectrum of (*S*)-1 at 1.0 × 10^−5^ M in the EtOH/H_2_O 10/90 mixture reveals a very weak dichroic response, presenting a small, positive bisignate Cotton effect with maxima at 570 nm (+) and 540 nm (−). These observations imply the presence of small aggregates with an excitonic coupling between monomers attributable to an H-type behaviour (Agg 0).^32,33^ AFM, TEM and SEM microscopies unravel the presence of ill-defined, globular aggregates of small dimensions (Fig. S2), a morphology that is characteristic of assemblies where hydrophobic interactions play a dominant role.^34^

**Fig. 2:**
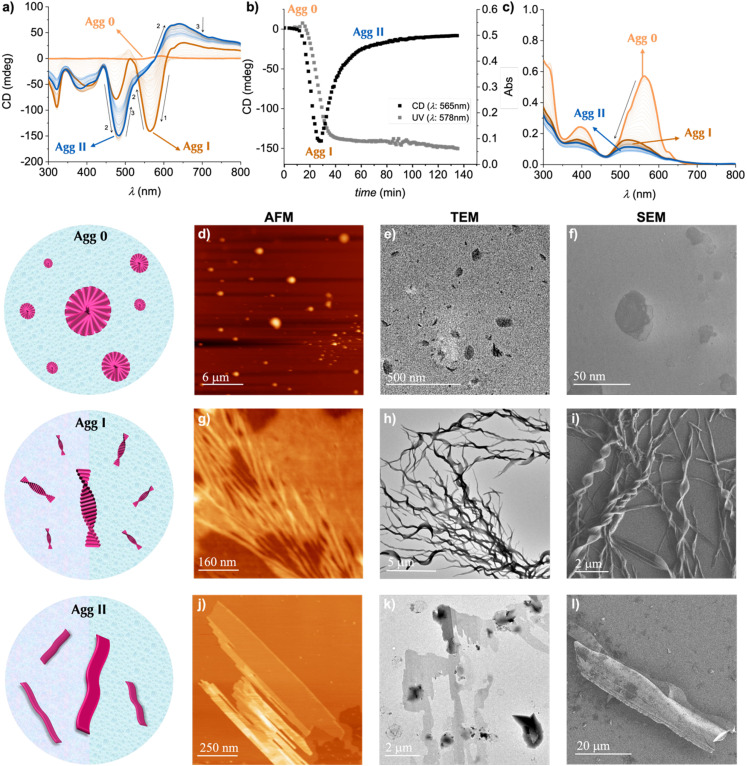
Self-assembly in solution of (*S*)-1 in EtOH/H_2_O 55/45 mixtures. (a) Time-dependent CD spectra showing the evolution of Agg 0 to Agg I and then to Agg II at 1.0 × 10^−5^ M (*l* = 1 cm, 20 °C). (b) Time evolution of the CD and UV-Vis signals after mixing equal volumes of the solution of (*S*)-1 in EtOH and (*S*)-1 in EtOH/H_2_O 10/90 mixtures at 1.0 × 10^−5^ M (*l* = 1 cm, 20 °C) (c) Time-dependent UV-Vis spectra showing the evolution from Agg 0 to Agg I and then to Agg II at 1.0 × 10^−5^ M (*l* = 1 cm, 20 °C). (d, g and j) AFM, (e, h and k) TEM and (f, i and l) SEM images of Agg 0 (upper panels), Agg I (middle panels) and Agg II (lower panels) formed by (*S*)-1 in EtOH/H_2_O 55/45 mixtures at 1.0 × 10^−5^ M.

During the screening of different EtOH/H_2_O mixtures, we observed a time-dependent evolution in the 55/45 mixture (Fig. 2a and S3, S4). Upon injecting equal volumes of a 1.0 × 10^−5^ M solution of Agg 0 formed by (*S*)-1 in EtOH/H_2_O 10/90 and the monomeric 1.0 × 10^−5^ M solution of (*S*)-1 in EtOH at 20 °C, the initial CD spectrum unveils a weak bisignate Cotton effect, similar to the previously observed weakly polymerized species attributed to Agg 0. However, after 15 minutes, a new chiroptical signature suddenly emerged, characterized by an intense positive bisignate Cotton effect with a maximum above 650 nm (+) and minimum at 560 nm (−), which we ascribe to the formation of a new aggregate (Agg I). This intermediate state then underwent a further slower transformation in the course of 40 min, resulting in a redshifted, non-symmetric positive bisignate Cotton effect, with bands above 600 nm (+) and 480 nm (−), denoting the formation of a second aggregate (Agg II). The rise of Agg II was accompanied by an increase in the CD signal and the optical density above 650 nm, together with the appearance of insoluble species. These two transitions are clearly observed when plotting the CD signal at 560 nm as a function of time (Fig. 2b, S4a and d). Interestingly, this type of behavior is exclusively discerned for the mixtures within a very narrow EtOH/H_2_O volume ratios, between 60/40 and 40/60. This emergence of distinct chiroptical properties in self-assembling π-conjugated systems within narrow compositions of polar organic solvents and water is quite unique. Pal *et al.* recently reported a similar system in mixtures of solvents like dioxane and DMSO with H_2_O percentages in the range of 40–60%.^35^

In UV-Vis spectroscopy, the changes exhibited with time by (*S*)-1 were also quite pronounced, with the main observations being a clear hypsochromic shift accompanied by an hypochromic effect and a red tailing above 600 nm, being this latter effect probably due to the partial precipitation of the sample (Fig. 2c, S4b and e).^36^ In fluorescence spectroscopy, the initial spectrum showed a maximum at 615 nm. Over time, this band increases in intensity and shifts to 605 nm – corresponding to the formation of Agg I – experiencing a subsequent decrease in intensity which we attribute to the emergence of Agg II, ultimately leading to insoluble species (Fig. S4c and f). These spectroscopic attributes could be in agreement with the formation of H-type aggregates with a slightly twisted intermolecular arrangement, as noted previously by other authors.^32,37^

To further investigate the morphological changes associated with the self-assembly process and given the large size of Agg II, AFM, TEM and SEM images were recorded at different time intervals after mixing the EtOH solution and the EtOH/H_2_O 10/90 solution to yield the EtOH/H_2_O 55/45 composition (Fig. 2d–l and further images in Fig. S5–S7). Immediately after mixing, helical fibrils with a thickness of 98.6 ± 9.7 nm were observed, likely corresponding to the formation of Agg I. Interestingly, these helices were mostly right-handed helices, which would be on agreement with the positive bisignate Cotton effect observed in CD spectroscopy, although this interpretation must be taken cautiously given the different sizes of the helices.^38^ After 20 minutes, the presence of helical fibrils became increasingly pronounced, as observed in the images taken at 20, 30, and 60 minutes, while the overall morphology remained similar. From this time onward, large precipitates started to appear, becoming visible at the macroscopic level. After 120 minutes, the sample predominantly consisted of 2D lamellar structures (Agg II), indicating extensive precipitation of the sample. At this stage, no fibrillar structures were detected in solution, implying that most of (*S*)-1 has flocculated and precipitated.

Altogether, these observations reveal that the self-assembly process in these conditions begins with the formation of helical fibrils (Agg I) from the preformed nuclei of Agg 0, which further interact and grow into Agg II, ultimately leading to precipitation and the formation of lamellar structures.

To gain deeper insight into these processes, we performed additional experiments using alternative preparation protocols, as well as variations in concentration, temperature of mixing and application of distinct cooling rates. Given the initial preparation protocol, it is plausible that Agg I and Agg II arise from Agg 0 rather than from the monomer. To test this hypothesis, we performed the mixing from a monomeric solution of (*S*)-1 at 2.0 × 10^−5^ M in EtOH with pure H_2_O, to yield the 1.0 × 10^−5^ M EtOH/H_2_O 55/45 solution of (*S*)-1. The formation of Agg I and Agg II occurred in an analogous manner to that of the previous preparation method, following even the same type of kinetics (Fig. S8). Performing the direct mixing from a monomeric solution (*S*)-1 at 1.0 × 10^−5^ M in EtOH with pure H_2_O to yield a 5.0 × 10^−6^ M EtOH/H_2_O 55/45 of (*S*)-1 only afforded Agg I, with no further evolution towards Agg II (Fig. S9). This concentration dependance would be in agreement with a hierarchical self-assembly process, *i.e.*, a consecutive pathway in which each stage involves self-assembled species of bigger dimensions.^39^ However, the increase of the optical density above 650 nm also suggests the partial precipitation of the sample during the process, being more accurate to describe this process as a self-assembly pathway on-route to non-classical crystallization.^11,12^ We additionally explored the direct mixing of a (*S*)-1 solution in EtOH and the (*S*)-1 EtOH/H_2_O 10/90 at 1.0 × 10^−5^ M at different temperatures, unveiling in all cases the final formation of Agg II (Fig. S10).

Finally, we also heated the (*S*)-1 solution in EtOH/H_2_O 55/45 to 70 °C to reach the monomeric state at the same concentration (1.0 × 10^−5^ M) and then we cooled it down at controlled rates (1 K min^−1^ and 5 K min^−1^) until different temperatures. Unfortunately, in most of these cases, this protocol resulted in a moderate CD signal and a loss of the absorption band due to the formation of a film of aggregates in the glass of the cuvette, as it occurs with other self-assembling π-conjugated systems.^40^ Although this band is relatively weak and this protocol does not seem to be optimal, the features observed in CD correspond to those of Agg II. The evolution of this signal with time seems to corroborate the formation of Agg II in the different conditions employed (Fig. S11). Therefore, due to these limitations, we could not further explore other experiments to corroborate these self-assembly processes.

Based on all these data, we hypothesize that after mixing, (*S*)-1 gradually grows from the nuclei into helical H*-*type aggregates (Agg I), which in turn further grow and interact to form chiral self-assembled structures of bigger dimensions and as will be shown below, higher degree of molecular order (Agg II). Over time, these Agg II structures collapse and precipitate, leading to the formation of insoluble 2D aggregates.

### Solid state studies

The propensity of (*S*)-1 to crystallize was inferred from the obtaining of macroscopic aggregates (Agg II) as a result of the self-assembly process together with preliminary studies in solid state by polarized optical microscopy (POM), which unambiguously unraveled the emergence of crystalline structures with micrometer sizes (Fig. S12).

**Fig. 3:**
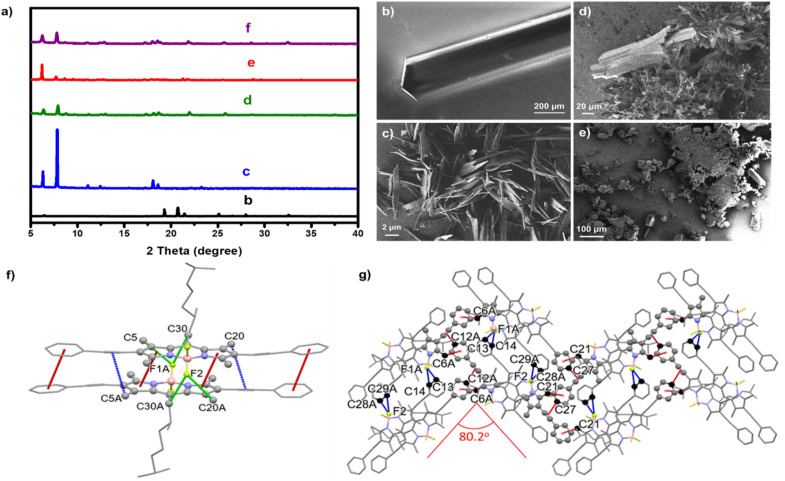
(a) Powder XRD diffractogram of (*S*)-1 samples prepared under different conditions: single crystal grown in acetonitrile/EtOH; (black trace); aggregates formed at 100 µM in EtOH/H_2_O mixtures at 100/0 (blue trace), 90/10 (green trace), 10/90 (red trace) and 45/55 (purple trace) volume ratios. (b–e) SEM images of self-assembled structures formed by (*S*)-1 at the conditions indicated in (a). (f) View of the dimer formed by the two molecules in the asymmetric unit of the (*S*)-1 derivative crystals. (g) Zig zag sheet extending in the *bc* plane, viewed along the *a*-axis. Intermolecular interactions are indicated by dashed lines in different colors: green for π–π; red for CH–π and blue for CH⋯F contacts. For clarity, the carbon atoms involved in these interactions are shown as grey spheres, and hydrogen atoms have been omitted. The structural parameters for non-covalent interactions are shown in Table S1.

To further explore the packing behavior of (*S*)-1 under different conditions, powder X-ray diffraction (p-XRD) measurements were performed on samples prepared in EtOH and EtOH/H_2_O mixtures with varying compositions (Fig. 3a). From those data, the distances corresponding to the main diffraction angles were calculated using Bragg's law (see SI, Section 2.2 and Tables S1–S4). The analysis reveals that (*S*)-1 undergoes a hierarchical self-assembly process in which local packing motifs emerge early and progressively develop into long-range ordered domains. In pure EtOH (blue trace in Fig. 3a), the diffractogram shows intense low-angle reflections (2θ ∼ 6–8°) corresponding to long *d*-spacings on the order of 14–11 Å, together with weaker higher-order reflections. This series of long periodicities is characteristic of a lamellar or layered supramolecular organization, in which (*S*)-1 forms regularly spaced domains with a defined interlayer repeat distance on the nanometer scale.^41,42^ As water is introduced (mixture EtOH/H_2_O 90/10, green trace in Fig. 3a), similarly intense low-angle reflections are discerned, attributable to several distinct long *d*-spacings (∼14, ∼11, ∼10 Å), and pointing out to the coexistence of multiple lamellar-like domains with slightly different periodicities.^42^ From these results it is inferred that partial enrichment of water promotes the formation of layered aggregates but also introduces heterogeneity in their interlayer spacing. At even higher water content (mixture EtOH/H_2_O 10/90, red trace in Fig. 3a), the diffractogram still displays pronounced low-angle features with large *d*-spacings, but these become broader and less well defined, consistent with rapid, solvophobic collapse into partially ordered lamellar fragments and a loss of long-range order,.^43^ which would on agreement with the spectroscopic and microscopic data observed for Agg 0 in these conditions. In all cases, however, weak reflections at higher diffraction angles (2*θ* ∼18–22°), corresponding to shorter *d*-spacings in the 5–4 Å range, are detected. These distances closely match the intermolecular separations resolved in the single-crystal X-ray structure of (*S*)-1, where antiparallel H-type dimers are stabilized by π–π, CH⋯F, and CH–π interactions and further organize into extended sheets (*vide infra*).^43^ The persistence of these 4–5 Å features, even in partially disordered aggregates formed under water-rich conditions, indicates that the same local packing motif present in the macroscopic single crystals is already established at much earlier stages of assembly. Taken together, these results support a hierarchical pathway in which (*S*)-1 first forms locally ordered chromophore dimers with crystal-like short-range packing, then arranges these dimers into lamellar superstructures with nanometer-scale periodicity whose regularity depends on solvent composition. When the conditions are favorable (absence of water or up to 1% in the precipitant), this supramolecular order can evolve into fully crystalline, micrometer-scale objects. SEM images supported these findings, showing that as the water content increased, the degree of crystallinity decreased (Fig. 3b–e).

To gain more insights in the nature of the ordered phases, p-XRD analysis was also performed for Agg II, obtained under the EtOH/H_2_O (55/45) conditions (purple trace in Fig. 3a and Table S5). The diffractogram of Agg II closely resembles that of the aggregate prepared in EtOH/H_2_O (90/10), displaying intense low-angle reflections (2*θ* ∼ 6–9°) corresponding to large *d*-spacings of 10–14 Å, characteristic of a lamellar or layered supramolecular organization in which molecular sheets are periodically separated by alkyl or solvent layers. Superimposed on this pattern, weaker peaks appear at higher diffraction angles (2*θ* ∼18–22°), associated with shorter intermolecular distances of 4–5 Å that match the π–π stacking and CH⋯F contacts observed in the single-crystal structure. Notably, the Agg II sample also exhibits faint reflections around 2*θ* ∼30°, a region characteristic of the crystalline phase, suggesting the presence of local domains with crystal-like order. These observations confirm that Agg II possesses a hybrid organization combining lamellar long-range order with local packing motifs analogous to those in the single-crystal lattice, providing direct experimental evidence of a crystallization pathway in which pre-organized aggregates act as structural precursors to macroscopic crystals.

Encouraged by these results, we attempted to grow larger crystals of (*S*)-1, suitable for single crystal analysis. Crystals were obtained by slow diffusion of an acetonitrile solution, with ethanol serving now as antisolvent. Interestingly, when crystals were grown under different conditions (such as slow evaporation in EtOH or slow diffusion in EtOH using an EtOH/H_2_O 99/1 mixture as a precipitant), the same space group and unit cell were obtained. It is worth noting that, although (*S*)-1 is soluble in EtOH at the concentrations required for self-assembly in solution (1.0 × 10^−5^ M), it becomes insoluble at higher concentrations (>1.0 × 10^−3^ M), which makes EtOH suitable for slow diffusion and facilitates the growth of high-quality crystals.

The bulk crystals showed sharp and well-resolved reflections in the powder XRD pattern (black trace), confirming their high crystallinity and long-range molecular order (Fig. 3a and b). Single crystal XRD analysis further revealed that (*S*)-1 crystallizes in the orthorhombic *P*2_1_2_1_2_1_ space group. The asymmetric unit contains two independent molecules, which exhibit slight differences in the orientation of the phenyl rings and alkyl chains. These molecules stack in a coplanar, arrangement, forming H-type dimers (Fig. 3f). Along the short axis, the dimers are disposed in a head-to-tail arrangement, with the BF_2_ groups oppositely oriented, and with a centroid-to-centroid angle of about 30°. Along the long axis, these BODIPY pairs are also slightly displaced with respect to each other. The dimers are stabilized by π–π stacking interactions involving both the pyrrole cores and phenyl substituents, which maintain an extended planar conformation, with an average centroid-to-centroid distance of ∼4.0 Å. The two (*S*)-1 units adopt an antiparallel configuration, with their BF_2_ groups oriented toward the methyl-pyrrolic hydrogen atoms and the C30 and C30A hydrogen atoms of the (*S*)-citronellol moieties, establishing CH⋯F interactions at 2.55 Å and 2.85 Å, respectively. Additionally, CH–π interactions are observed between the methyl-groups at the pyrrole units and the alkyne moieties of adjacent molecules, further contributing to the packing stability. The three-dimensional arrangement consists of zigzag sheets extended in the *bc* plane (Fig. 3g), characterized by an intermolecular angle of 80.2°. Along the *c*-axis, dimer association is mediated by CH⋯π interactions between the methyl groups of the pyrrole rings and the phenyl substituents. Meanwhile, the *para*-hydrogen atoms of the phenyl rings engage in interactions with the alkyne groups of neighboring molecules. Along the *b*-axis, the dimers are connected through additional intermolecular CH⋯F interactions, contributing to the overall stabilization of the crystal packing. All in all, these interactions indicate that H-type dimers act as the primary aggregation units, which further organize into higher order structures along the *b*-axis through interdimer CH⋯F interactions (Fig. S13).

Fluorescence spectroscopy of the crystals revealed a red-shifted emission band centered at 685 nm (*λ*_exc_ = 530 nm), similar to the spectral features observed for Agg II in solution although at longer wavelengths (Fig. S14a). The excitation spectrum recorded at 685 nm emission displayed three distinct bands centered at 341, 463, and 632 nm, which are also red shifted compared to the absorption spectra in solution, however sharing the same spectroscopic features (Fig. S14b). These consistent red-shifts, of about ∼60 nm, can be tentatively attributed to diverse reasons. Firstly, the chemical environments and techniques employed in the crystal *versus* solution samples are different. Secondly, the structure of the H-type dimers is not perfectly coplanar, but show small, centroid-to-centroid angles and a head-to-tail arrangement along the short axis. In addition, some kind of J-type excitonic coupling could be inferred from the single crystals, although the distances observed between the dimers make it unlikely. Lastly, a chief contributor for the red shifts observed may be the considerable planarization of the entire π-system observed in the crystal, which deactivates rotational motions around the σ-bonds and thus enhances π-conjugation with respect to dissolved BODIPY molecules. Notably, varying the excitation wavelength among these bands did not alter the position of the emission maximum, which consistently remained at 685 nm. The fluorescence quantum yield in the solid state was determined to be 5%, which is lower than that measured in solution but remarkable for a crystalline structure. Although structurally similar BODIPY derivatives have been reported to exhibit quantum yields up to 9%,^24^ these values were obtained from thin films, making therefore direct comparisons unreliable. Furthermore, while some solid-state crystalline BODIPY derivatives have achieved fluorescence quantum yields as high as ∼32% in the red region (typically 601–632 nm),^44^ such high efficiencies are observed at shorter wavelengths than in our case. In contrast, our system emits at 685 nm, approaching the deep-red/NIR region, where fluorescence efficiency is generally reduced due to increased non-radiative decay (as predicted by the energy-gap law).^45^ Therefore, a solid-state quantum yield of 5% at 685 nm is not only noteworthy, but also indicative of a favorable molecular arrangement that preserves emission despite the long-wavelength regime.

### Optical waveguide behavior

The hierarchical self-assembly of (*S*)-1 culminates in macroscopic crystalline aggregates (Agg II) with a high degree of molecular order, essentially mirroring the packing motif observed in the single-crystal XRD structure. This structural continuity between solution-grown aggregates and bulk crystals suggests that the ordered arrangement achieved through self-assembly could translate into enhanced optoelectronic functionality. In particular, the final aggregates exhibit intense deep-red photoluminescence (*λ*_em_ = 685 nm), having a good quantum yield value (5%) despite emitting in the near-IR. The combination of strong fluorescence and minimal disorder (as indicated by the low defect density in the crystals) is a hallmark of high-quality organic materials.^19^ Recognizing these favorable attributes, we sought to examine whether the self-assembled crystals of (*S*)-1 could actively transport light as optical waveguides, thereby translating our structural findings to a tangible photonic function. Numerous organic crystals with waveguide behavior have been reported in the last years, mainly in the UV-visible range, with only few examples approaching the NIR range.^46,47^

Several known factors can influence light propagation in organic crystalline waveguides, and our system inherently satisfies many of these criteria. Efficient waveguiding generally requires materials with high intrinsic luminescence and few crystallographic defects to minimize optical losses, requirements that are met by (*S*)-1 crystals. Additionally, recent studies report that the presence of internal microchannels within an organic crystal can reduce scattering and enhance waveguiding performance by an exciton–polariton mechanism.^48,49^ Our single-crystal analysis uncovered one-dimensional channels running through the (*S*)-1 lattice, also apparent in projections of the packing structure (Fig. S15). This unique architecture, together with the material's high luminescence and structural integrity, strongly supports the suitability of (*S*)-1 crystals for efficient light guiding.^18^

**Fig. 4:**
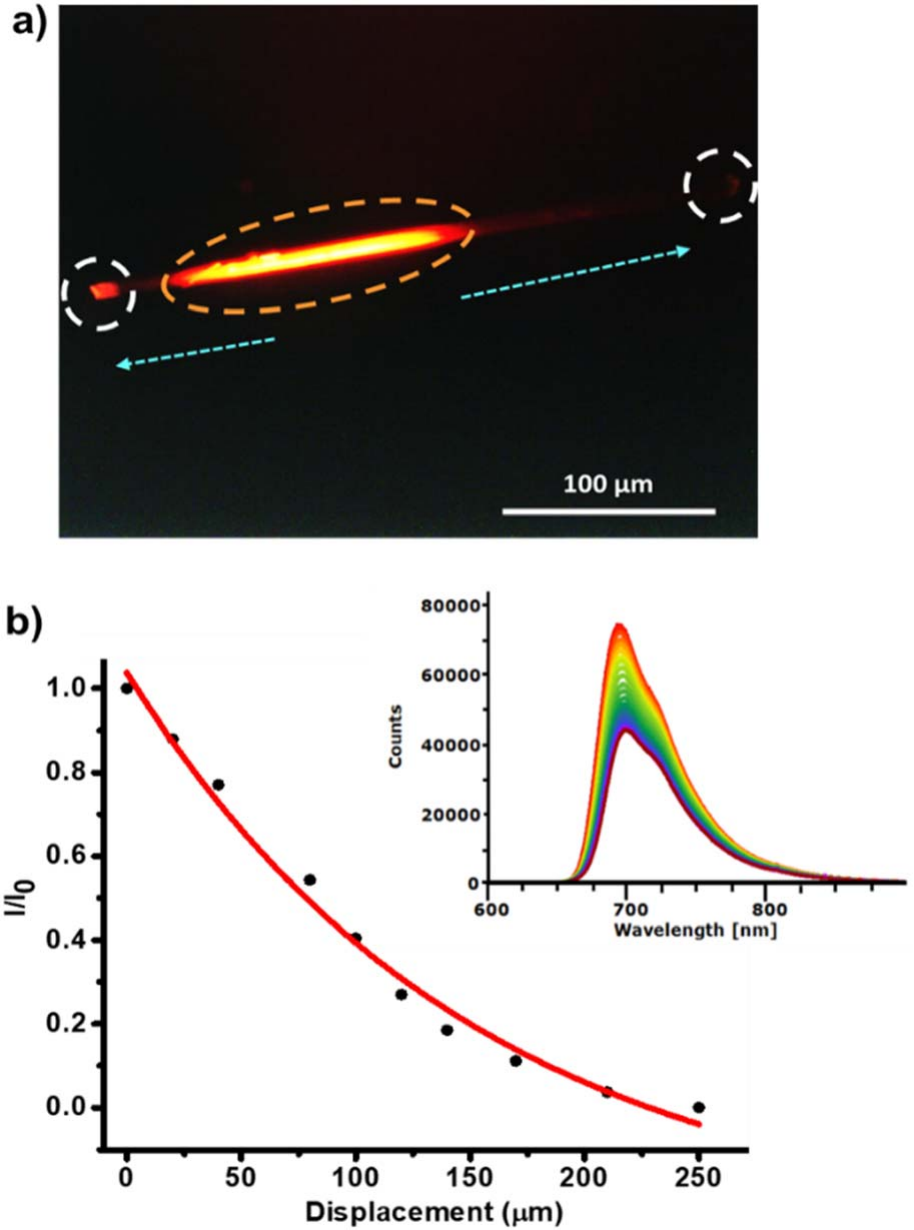
(a) PL microscopy image of the (*S*)-1 single crystal. The orange circle indicates the excitation point, the white circles are the emission outputs, while the light propagation direction is indicated by the cyan arrows. (b) Ratio between the PL intensities photoexcited at the initial (*I*_0_) and intermediate (*I*) positions along the crystal as a function of distance. The red line corresponds to the fit to exponential function dictated by Lambert–Beer law (*λ*_exc_ = 355 nm). PL spectra of the crystal collected at the end tip upon varying the distances between the excitation and the tip of crystal (each spectrum in a different color was registered at a different distance).

To qualitatively assess the optical waveguiding behavior, photoluminescence (PL) microscopy images were recorded (Fig. 4a). Upon localized photoexcitation at 355 nm, the crystals exhibited intense emission at 698 nm at one end, with negligible emission along the crystal body, a behavior that is characteristic of efficient active waveguiding materials. To quantify the waveguiding efficiency, the optical loss coefficient was determined by shifting the photoexcitation spot along the crystal length while monitoring the emission at one tip. The intensity of the emitted light (*I*_out_) at a distance *x* from the excitation site (*I*_in_) follows the Lambert–Beer law *I*_out_ = *I*_in_*e*^−*αx*^, where *α* is the absorption coefficient (µm^−1^). The optical loss coefficient (*α*_0_, in dB µm^−1^) is related to *α* by the expression: *α*_0_ = 4.34 *α*.

For the (*S*)-1 crystals, *α* and *α*_0_ were determined to be 0.037 µm^−1^ and 0.016 dB µm^−1^, respectively (Fig. 4b). To our knowledge, this is the first reported quantitative measurement of the optical loss coefficient for a BODIPY-based crystal used as an optical waveguide. Although this *α*_0_ value is not the lowest reported among organic waveguiding crystals, it is well within the range of the most efficient organic systems,^50,51^ highlighting the promising performance of this and related BODIPY materials.

## Conclusions

Herein, we have investigated the self-assembly processes of the chiral BODIPY derivative (*S*)-1 across scales, from the monomer to diverse aggregated states in solution that evolve to crystalline structures in the solid state. The study of its self-assembly behavior in EtOH/H_2_O mixtures revealed a time-dependent self-assembly process reliant on the preparation method and solvent composition, ultimately leading with time to highly ordered aggregates with propensity to precipitate from which the crystallinity of (*S*)-1 could also be inferred. The subsequent growth of single crystals permitted us to correlate the supramolecular organization in solution to that observed in the solid state. Importantly, the resulting long crystals presented efficient active optical waveguiding behavior, constituting the first quantitative optical loss reported for BODIPY crystals. These findings underscore the potential of this important class of photoactive π-conjugated systems in organic photonics applications. Overall, this work highlights the importance of a comprehensive, multiscale understanding of self-assembly processes, understanding the transition from the monomer to the material, and demonstrating how such insight has enabled us to expand the functional scope of BODIPY derivatives in the field of organic electronics.

## Author contributions

M. G. S. synthesized and characterized the molecules and carried out the supramolecular studies in solution as well as the TEM and AFM microscopy experiments. A. M. G. prepared the crystals, performed the solid-state experiments, and conducted the SEM analysis. J. S. V. wrote the manuscript and performed the supramolecular experiments in solution. A. M. R. conducted the crystallographic analysis. J. C.-G. carried out the photoluminescence measurements. P. P. and D. G. R. conceived and supervised the project. All authors contributed to the editing and revision of the manuscript.

## Conflicts of interest

There are no conflicts to declare.

## Supplementary Material

SC-017-D5SC07776A-s001

SC-017-D5SC07776A-s002

## Data Availability

CCDC 2476985 contains the supplementary crystallographic data for this paper.^52^ All data supporting the findings of this study are provided in the supplementary information (SI). Supplementary information: experimental procedures, optimisation data, mechanistic studies, and NMR spectra. See DOI: https://doi.org/10.1039/d5sc07776a.
