# Supplementary material for: Understanding self-assembly across scales in BODIPY derivatives: from supramolecular aggregation in solution to optical-waveguiding crystalline materials
Source: Chem Sci. 2025 Nov 28;17(4):2386–94. doi: 10.1039/d5sc07776a (PMC12684744; doi:10.1039/d5sc07776a)
Supplement: SC-017-D5SC07776A-s001 [file SC-017-D5SC07776A-s001.pdf]

Supplementary Information (SI) for Chemical Science.
This journal is © The Royal Society of Chemistry 2025

SUPPORTING INFORMATION

Understanding self-assembly across scales in BODIPY derivatives: from supramolecular aggregation in solution to optical waveguiding crystalline materials

Marina González-Sánchez, Ana M. García, Jorge S. Valera, Ana M. Rodríguez, Juan Cabanillas, Pilar Prieto * and David González Rodríguez*

Materials and methods

All reagents and solvents were obtained from commercial suppliers and used without further purification. Solvents for spectroscopic studies were of spectroscopic grade and used as received. Column chromatography was carried out on silica gel Merck-60 (230-400 mesh, 60 Å), and TLC on aluminum sheets precoated with silica gel 60 F254 (Merck).

NMR experiments. ^{1}H-NMR, ^{13}C-NMR were recorded with a BRUKER AVANCE-II 300 MHz or a BRUKER DRX 500 MHz instrument. The temperature was actively controlled at 298 K. Chemical shifts are measured in ppm using the signals of the deuterated solvent as the internal standard [$CDCl_3$ calibrated at 7.26 ppm (^{1}H) and 75.0 ppm (^{13}C)]. Coupling constants (*J*) are denoted in Hz and chemical shifts (δ) in ppm. Multiplicities are denoted as: s = singlet, d = doublet, t = triplet, m = multiplet, br = broad.

Mass Spectrometry (MS) and High Resolution-Mass Spectrometry (HRMS) MALDI-TOF spectra were obtained from a BRUKER ULTRAFLEX III instrument equipped with a nitrogen laser operating at 337 nm.

UV-Vis spectroscopy: Measurements in solution were conducted using a JASCO V-660 apparatus.

Fluorescence spectroscopy: Measurements in solution were performed JASCO FP-8600 equipment using excitation and emission bandwidths of 5 nm in both cases, and a 50 ms response. Measurements in the solid state were performed on a Jasco FP-8300 spectrophotometer, using the FDA-808 solid sample holder, with the crystals deposited on a quartz slide place on the holder. Collected data were plotted using OriginPro 9.1. Photoluminescence quantum yields in solution were calculated relative to quinine sulphate in sulphuric acid 0.1 M (ΦPL = 0.54) as the reference. Photoluminescence quantum yields of solid samples (crystals) were determined using the same spectrophotometer incorporating a Jasco ILF-835/100 mm integrating sphere.

CD spectra were recorded with a JASCO J-815 equipment (measurement information: data pitch = 1 nm; sensitivity = standard; D.I.T. = 2 sec; slit width = 1000 [illegible]m; data interval = 1 nm; scanning speed = 200 nm/min). In all these three instruments the temperature was controlled using a JASCO Peltier thermostatted cell holder with a range of 263–383 K, adjustable temperature slope, and accuracy of ± 0.1 K.

Transmission electron microscopy (TEM) images were obtained JEOL JEM 1400 PLUS instrument operating at an accelerating voltage of 40 to 120 kV, which provided high contrast and resolution. Sample solutions were deposited on 200 mesh formvar copper grids coated with carbon

Atomic Force Microscopy (AFM) images were obtained with a multimode Nanoscope III A Bruker instrument (Infraestructura Científico Técnica Singular, ICTS; Centro Nacional de Microscopía Electrónica, CNME). The samples were spin-coated on HOPG or mica. Samples were deposited onto a glass substrate and left to dry under vacuum or in air, covered from light.

Scanning Electron Microscopy (SEM): SEM images were obtained on a JEOL JSM 6335F microscope working at 10 kV. The samples for SEM imaging were prepared by deposition onto

a glass substrate, removing the excess of solvent with filter paper and drying the sample in vacuum or in air, and covered from light.

Fluorescence microscopy: Fluorescence imaging was performed with a Zeiss Axio Vert A.1. microscope, using 10x and 20x objectives, and different filters.

Single crystal X-ray Diffraction analysis for (S)-1, CCDC number 2476985 Crystal evaluation and data collection were performed at room temperature on a Bruker X8 APEXII CCD area detector diffractometer using graphite monochromated Mo-Kα radiation (λ = 0.71073 Å). The datasets were integrated with SAINT[1] and corrected for Lorentzian and polarization effects. A semi-empirical absorption correction was applied to the diffraction data using SADABS.[2] The software package OLEX2[3] was used for structure solution and refinement by full-matrix least-squares methods based on F.[2] A successful solution by direct methods provided most non-hydrogen atoms from the E map. The remaining non-hydrogen atoms were located in an alternating series of least-squares cycles and different Fourier maps. The non-hydrogen atoms were refined with anisotropic displacement coefficients and hydrogen atoms were placed by using a riding model and included in the refinement at calculated positions. The crystal exhibits very weak diffraction and the Flack parameter could not be determined from the experimental data, so the same chirality as that of the reagents used has been assigned. The distances and angles for the non-covalent interactions (hydrogen interactions, π stacking, etc.) were evaluated using the program Mercury.[4]

Powder X-ray Diffraction (p-XRD) analysis. p-XRD patterns for **(*S*)-1** samples were recorded on a Bruker D8 Advance A25 diffractometer equipped with a vertical goniometer in a θ/θ configuration using Cu Kα radiation. The instrument is fitted with variable divergence slits on both the primary and secondary beams for Bragg–Brentano geometry, and a Lynxeye XE-T detector, an ultrafast, energy-dispersive, high-resolution detector that functions as a built-in monochromator. Solutions of **(*S*)-1** were prepared in EtOH and EtOH/H_2O mixtures with varying ratios. The samples were allowed to stand for 60 minutes, then deposited on the sample holder (solutions or precipitates obtained from solutions) and left to dry. For the single crystals, the materials were gently homogenized with a spatula to obtain fine powders, which were then placed on the sample holder. Diffraction data were collected over a suitable 2θ range, and the resulting patterns were processed and plotted using Origin 9.1 software.

Optical waveguide behaviour: PL microscopy images for crystal needles were acquired with a Nikon Eclipse Ti inverted microscope with dry objectives (100X N.A. 0.8 and 20X N.A. 0.45) coupled to a Shamrock spectrometer from Andor Technology with a thermoelectrically cooled Newton EM (Andor) CCD. The excitation was obtained by appropriate filtering of the lines from a Xe lamp.
Loss coefficients in crystals were obtained upon exciting them with a pulsed Nd:YAG laser (355 nm, 300 ps, 1 KHz, 30 mJ/pulse). A set of filters were employed to attenuate the photoexcitation. Detection from the crystal edge was focused in free space on to a 0.5 m length SP2558 Princeton Instruments (Acton Research) spectrometer equipped with a 600 lines/mm grating and a liquid nitrogen cooled CCD.

0. Synthetic procedures and characterization data

The inclusion of the chiral center in the BODIPY core was achieved through the condensation of an acid chloride obtained from (*S*)-citronellol with the corresponding 2,4-dimethylpyrrole, followed by subsequent reaction with $BF_3.OEt_2$ in the presence of NEt_3. After iodination of the chiral BODIPY core with NIS, the final step involves a Sonogashira type coupling between this scaffold and ethynyl benzene, yielding **(*S*)-1** in moderate yields

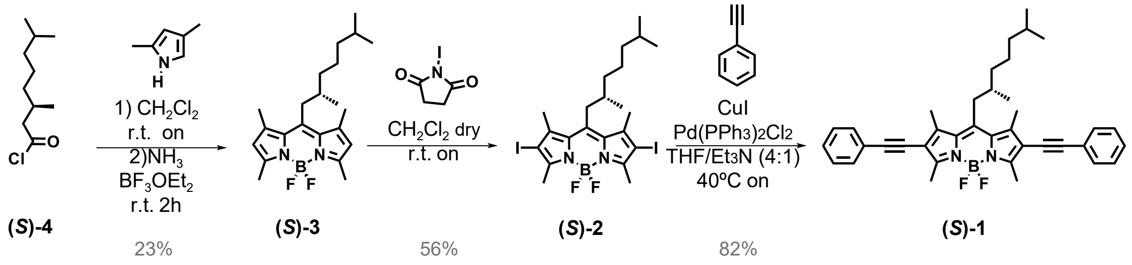

Scheme S0A. Synthetic route of the synthesis of **(*S*)-1**. **(*S*)-4** was obtained through a previously described method and presented the spectroscopic features reported therein.[5]

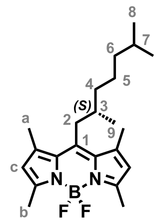

(*S*)-3. A mixture of **(*S*)-4** (500 mg, 1 eq, 2.6 mmol) in a flask under argon atmosphere was dissolved in dry CH_2Cl_2 (50 ml) and previously distilled 2,4-dimethyl-pyrrole was added (500 mg, 2 eq, 5.26 mmol). The reaction mixture was stirred overnight at room temperature. After that time, NEt_3 (2.3 ml, 6.2 eq, 16.3 mmol) and BF_3OEt_2 (2.3 ml, 7 eq, 18.4 mmol) were added and after 2 hours the resulting crude was washed with water. The organic layer was extracted and dried with $MgSO_4$. After filtering and removal of the solvent, the resulting crude was purified by column chromatography, using the mixture cyclohexane: ethyl acetate (100:1) as eluent, affording **(*S*)-3**. as an orange-red solid (540 mg, 23%). **^{1}H-NMR** (300 MHz, $CDCl_3$): δ = 6.05 (s, 2H, H^c), 2.94 (m, 2H, H^2), 2.52 (s, 6H, CH_3^b), 2.42 (d, *J* = 5.8 Hz, 6H, CH_3^a), 1.33 – 1.07 (m, 8H, $CH^{3,7}$, $CH_3^{4,5,6}$), 0.98 – 0.80 (m, 9H, $CH_3^{8,9}$). **^{13}C-NMR** (75 MHz, $CDCl_3$): δ = 146.5, 76.5, 39.0, 37.5, 36.9, 34.8, 31.9, 31.4, 30.2, 29.7, 29.3, 28.0, 27.1, 25.0, 22.7, 22.5, 18.4, 17.5, 16.8, 14.4, 14.1. **MS** (MALDI$^+$, matrix DCTB): Calculated for $C_{22}H_{33}BF_2N_2$ $[M]^+$: 374.3. Found $[M]^+$: 374.3.

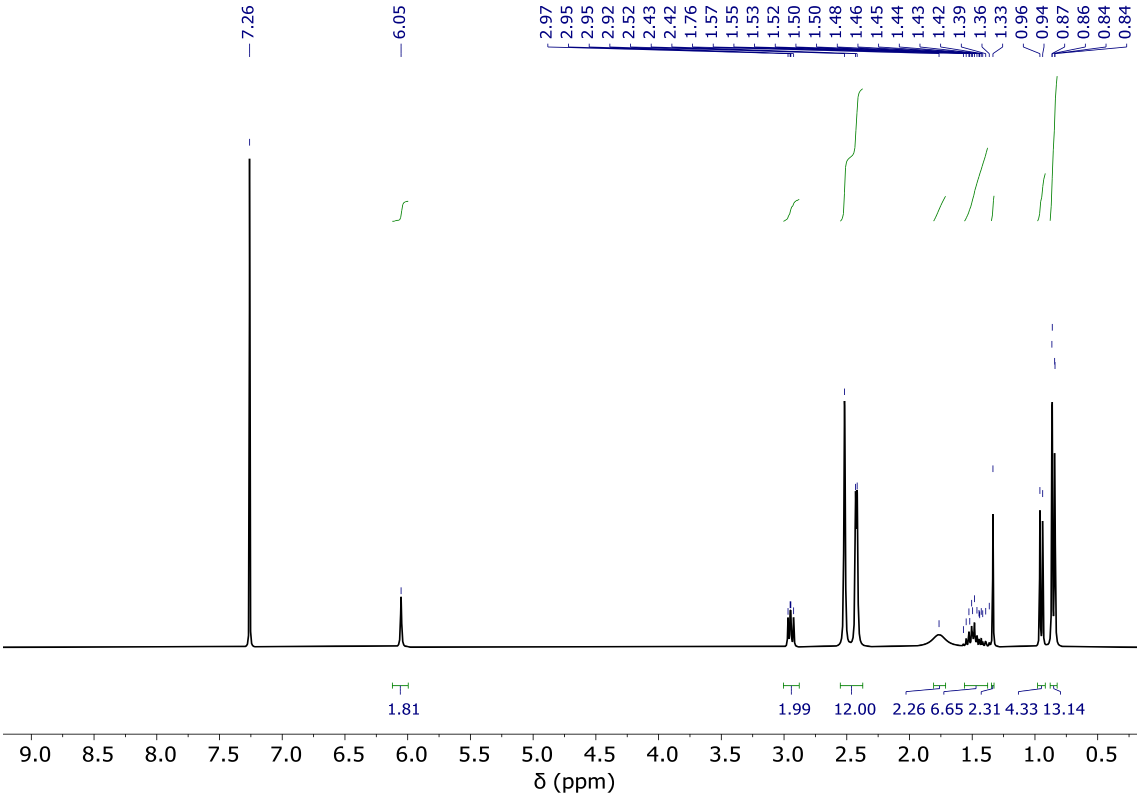

Figure S0A. ^{1}H NMR spectrum of ***(S)*-3** (300 MHz, $CDCl_3$)

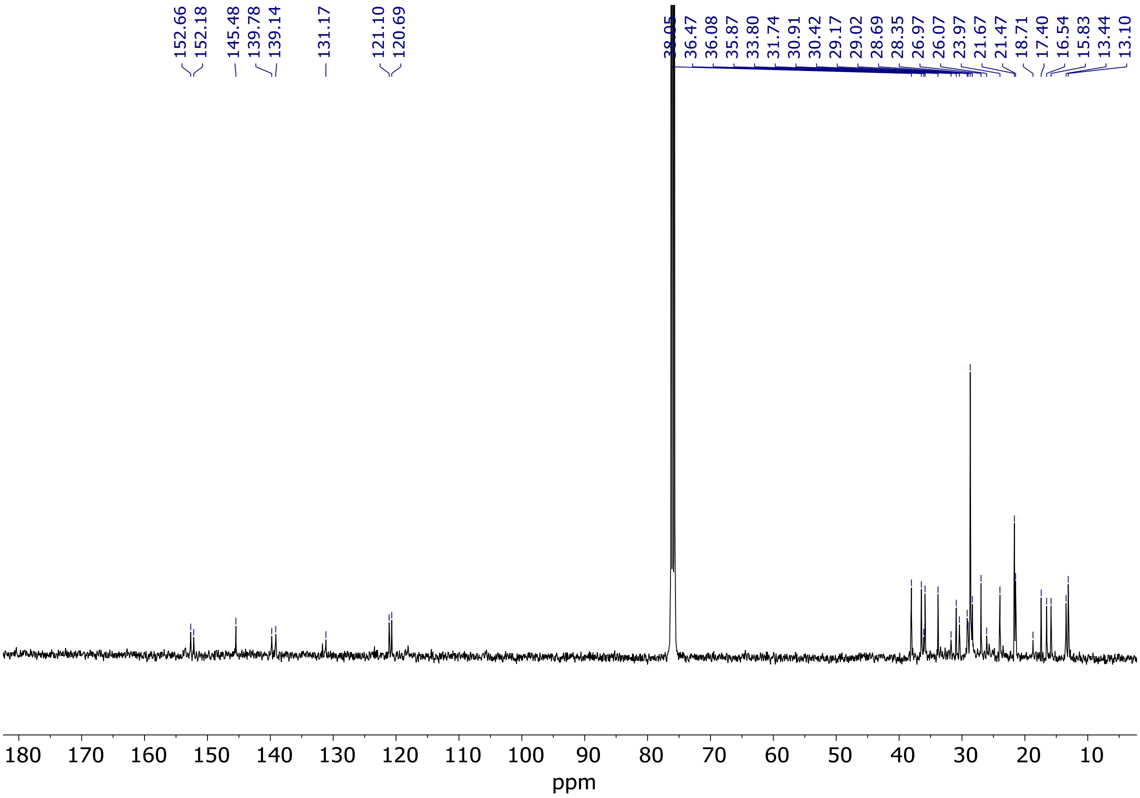

Figure S0B. ^{13}C NMR spectrum of ***(S)*-3** (75 MHz, $CDCl_3$)

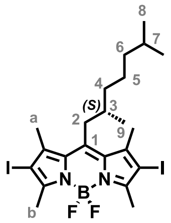

(*S*)-2. To solution of **(*S*)-3** (150 mg, 1 eq, 0.4 mmol) in dry CH_2Cl_2 was added dropwsise a solution of NIS (225 mg, 2.5 eq, 1 mmol) in dry DMF under argon atmosphere. The mixture was stirred vigorously overnight at room temperature. Once the reaction was finished the mixture was washed with water and brine. The organic layer was dried over $MgSO_4$ and the solvent was evaporated under reduced pressure. The product was purified by column chromatograph using the mixture cyclohexane:ethyl acetate (5:1) as eluent, yielding **(*S*)-2** as an orange-red solid (144 mg, 56%). **^{1}H-NMR** (300 MHz, $CDCl_3$): δ = 2.97 (m, 2H, CH_2^{2}), 2.54 (s, 6H, CH_3^{b}), 2.41 (d, *J* = 5.8 Hz, 6H, CH_3^{a}), 1.66 (s broad, 1H, CH^{3}) 1.52 – 1.33 (m, 4H, $CH_2^{4,5}$), 1.19 (m, 3H, CH^{7}, CH_2^{6}), 0.85 (d, *J* = 6.7 Hz, 3H, CH_3^{9}), 0.78 (d, *J* = 6.7 Hz, 6H, CH_3^{8}). **^{13}C-NMR** (75 MHz, $CDCl_3$): δ = 155.1, 154.7, 146.5, 142.6, 142.0, 132.5, 131.7, 87.1, 86.5, 39.0, 37.5, 37.1, 35.6, 28.0, 24.9, 22.6, 20.2, 19.5, 18.4, 16.1. **MS** (MALDI+, matrix DCTB): Calculated for $C_{22}H_{32}BF_2I_2N_2$ $[M+H]^+$: 626.1 Found $[M+H]^+$: 626.1.

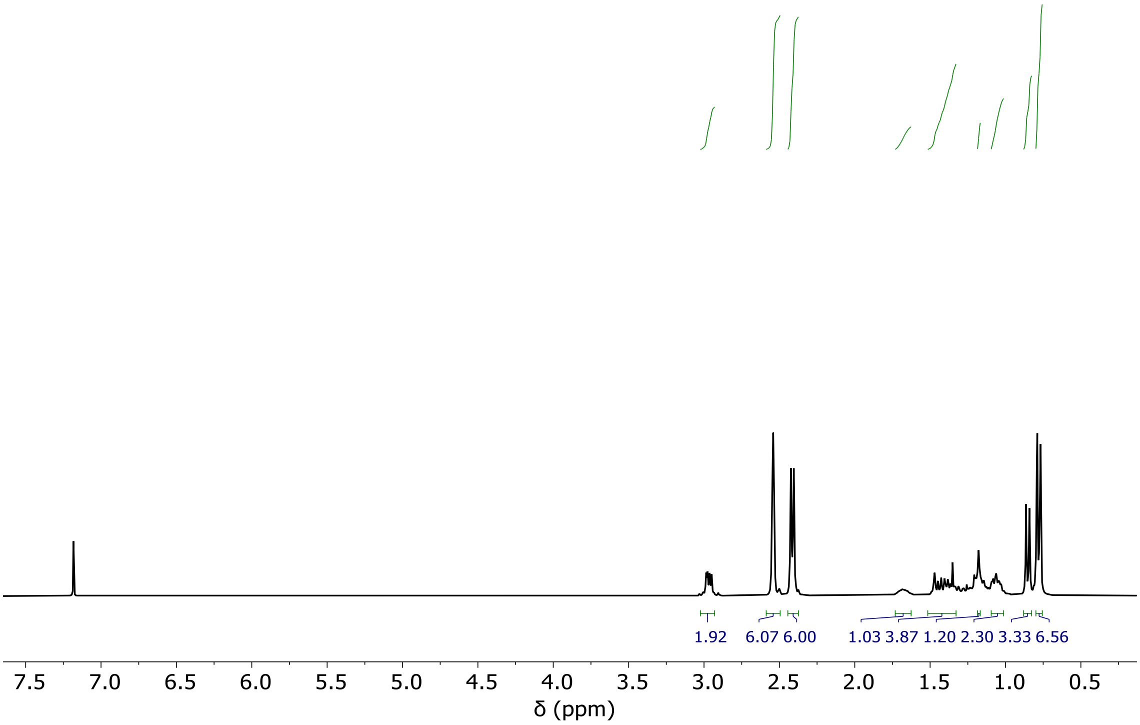

Figure S0C. ^{1}H NMR spectrum of **(*S*)-2** (300 MHz, $CDCl_3$)

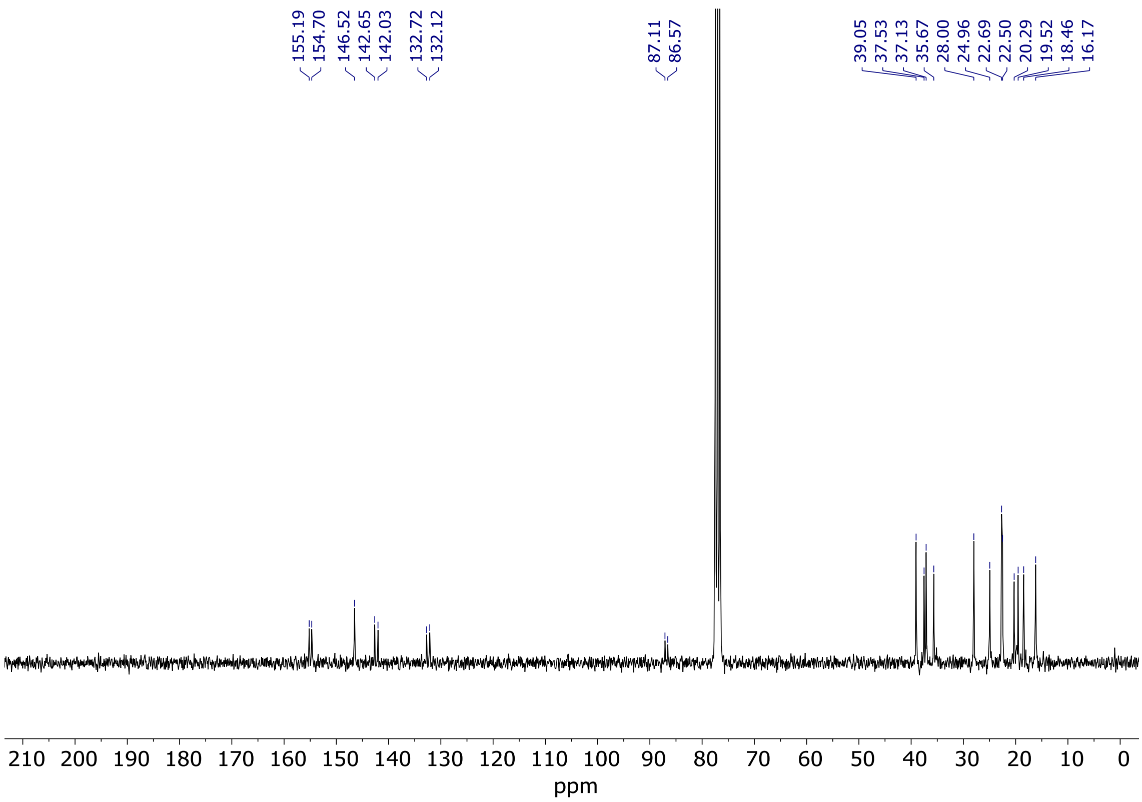

Figure S0D.^{13}C NMR spectrum of ***(S)*-2** (75 MHz, $CDCl_3$)

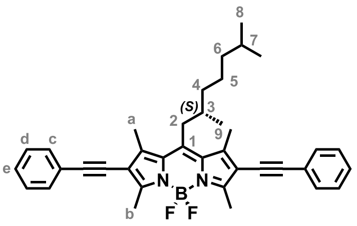

(*S*)-1. A dry THF/NEt_3 (4:1) mixture (150 mL) was subjected to deoxygenation by three freeze-pump-thaw cycles with argon. It was then poured over a round-bottom flask containing **(*S*)-2** (1.0 g, 1 eq, 2.3 mmol), $Pd(PPh_3)_2Cl_2$ (32 mg, 0.02 eq, 45.6 µmol), and CuI (4.3 mg, 0.01 eq, 22.8 µmol). Subsequently, phenylacetylene (2.4 g, 2.5 eq, 5.7 mmol) was added dropwise. The resulting mixture was stirred under argon atmosphere at 40 ºC. After checking completition by TLC, the mixture was filtrated over a celite plug and the solvent was evaporated under reduced pressure. The resulting residue was purified by silica gel column chromatography using ethyl acetate:$CHCl_3$ as eluent to give **(*S*)-1** as a pinkish solid (1.93 g, 82%). **^{1}H-NMR** (300 MHz, $CDCl_3$): δ = 7.54 -7.49 (m, 4H, CH^c), 7.38 – 7.32 (m, 6H, CH^d, CH^e), 3.09 – 3.03 (m, 2H, CH_2^2), 2.69 (s, 6H, CH_3^b) 2.63 – 2.57 (d, *J* = 6.7 Hz, 6H, CH_3^a), 1.64 (m, 1H, CH^3) 1.53 (m, 2H, CH_2^4), 1.42 (m, 2H, CH_2^5), 1.37 (m, 1H, CH^7), 1.26 (m, 2H, CH_2^6), 0.99 – 0.95 (d, *J* = 6.6 Hz, 3H, CH_3^9), 0.88 – 0.84 (dd, *J* = 6.6, 1.2 Hz, 6H, CH_3^8). **^{13}C-NMR** (75 MHz, $CDCl_3$): δ = 156.3, 155.9, 147.1, 141.1, 140.5, 131.6, 130.8, 127.9, 127.6, 122.9, 96.0, 81.4, 76.9, 76.7, 76.5, 76.1, 42.9, 38.5, 37.0, 36.6, 34.8, 31.4, 29.7, 29.2, 27.5, 26.4, 24.5, 22.2, 22.0, 18.0, 15.9, 15.2, 13.6, 13.1. HR**MS** (APCI+): Calculated for $C_{38}H_{41}BF_2N_2$ $[M]^+$: 573.3362. Found $[M]^+$: 573.3365.

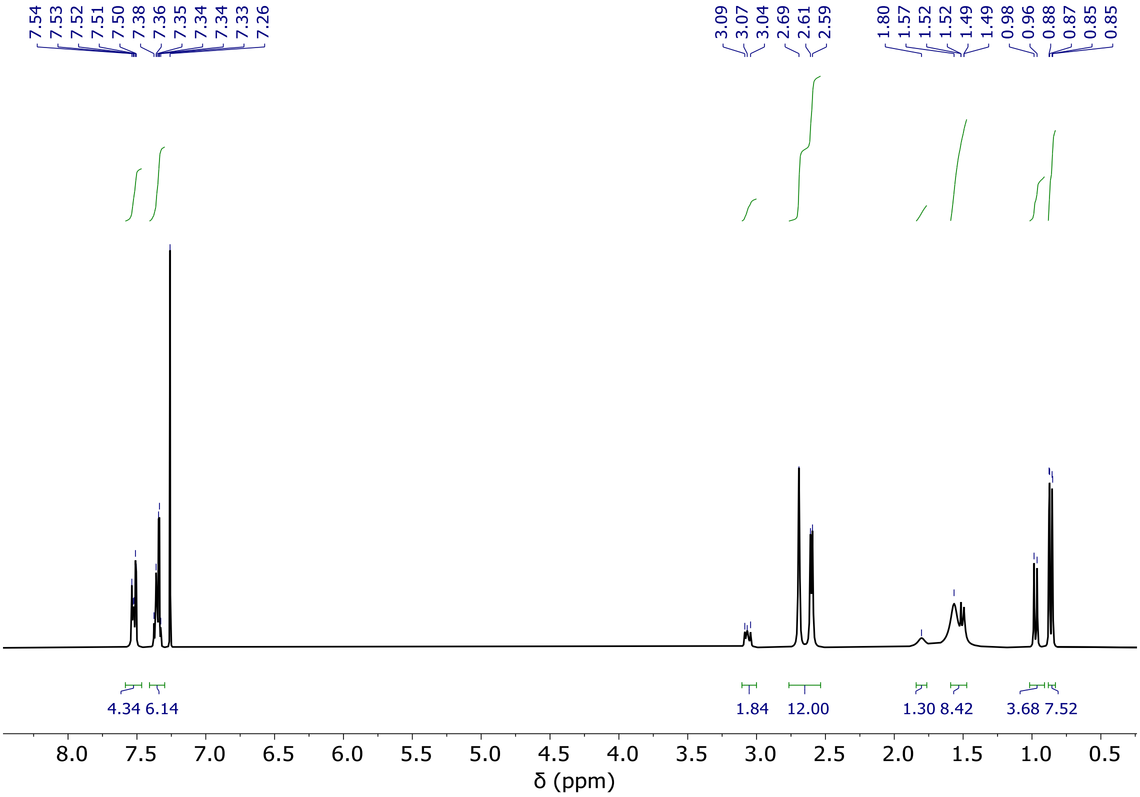

Figure S0E.^{1}H NMR spectrum of ***(S)*-1** (300 MHz, $CDCl_3$)

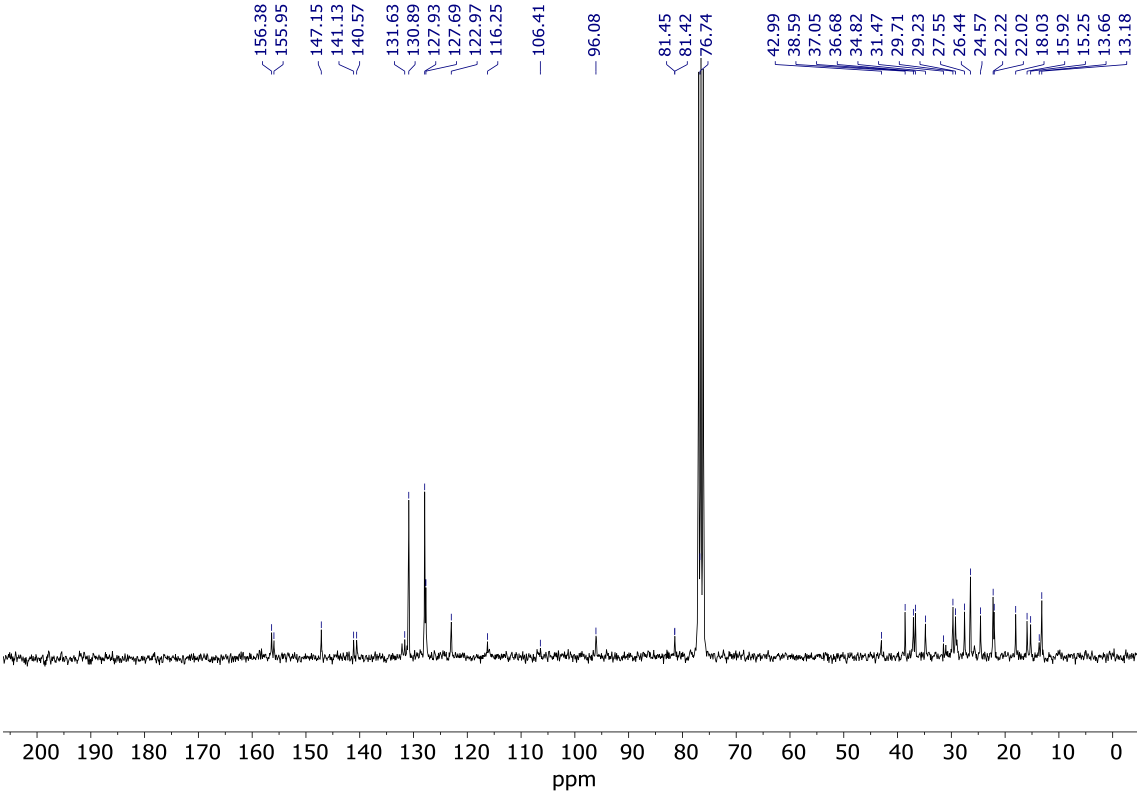

Figure S0F.^{13}C NMR spectrum of ***(S)*-1** (75 MHz, $CDCl_3$)

1. Self-assembly studies in solution

General Remarks. A series of spectroscopic experiments were performed with **(*S*)-1** with the aim to demonstrate the equilibrium between the monomer and the self-assembled polymer(s) in solution, as well as the influence of the preparation protocol in these phenomena. We explored the effects of solvent, temperature, and kinetics through different spectroscopic and microscopic techniques.

1.1 Solvent-dependent experiments

Firstly, we assessed the self-assembly behavior of **(*S*)-1** by using different solvents (EtOH, EtOH-H_2O 10/90, heptane, MeCN, and THF). Figure S1 shows that **(*S*)-1** in organic solvents exhibit notable differences in absorption, fluorescence emission and CD experiments compared to those showcased in aqueous medium. The absorption spectra (Figure S1a) unveil a significant broadening of the absorption band, and the fluorescence emission results (Figure S1b) unravel a red shift from 620 to 625 nm and a significant quenching of the sample signal in aqueous medium compared to the organic medium. In CD spectroscopy (Figure S1c) the slight Cotton effect with maxima at 540 nm turns into a moderate and positive bisignate Cotton effect with maxima at 585 nm (+) and 520 nm (-).

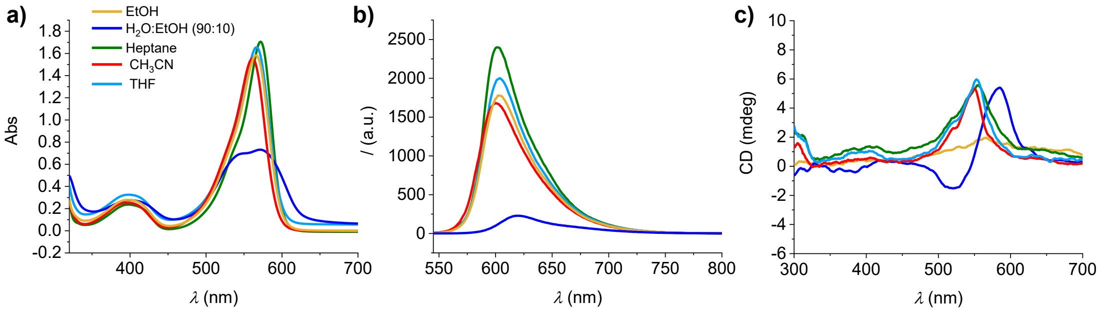

Figure S1. (**a**) UV-Vis, (**b**) fluorescence ($\square_{exc}$ = 550 nm) and (**c**) CD spectra of **(*S*)-1** in EtOH (yellow lines), EtOH/H_2O (10/90) mixtures (dark blue lines), heptane (green lines), acetonitrile (red lines) and THF (light blue lines). [**(*S*)-1**] = 1.0·10^{-5} M, (20 ºC, l = 1 cm for all experiments).

The characterization by different microscopic techniques (AFM, TEM and SEM) of the aggregates observed in EtOH-H_2O 10/90 discloses the presence of ill-defined nanostructures in both conditions, as seen in Figure S2.

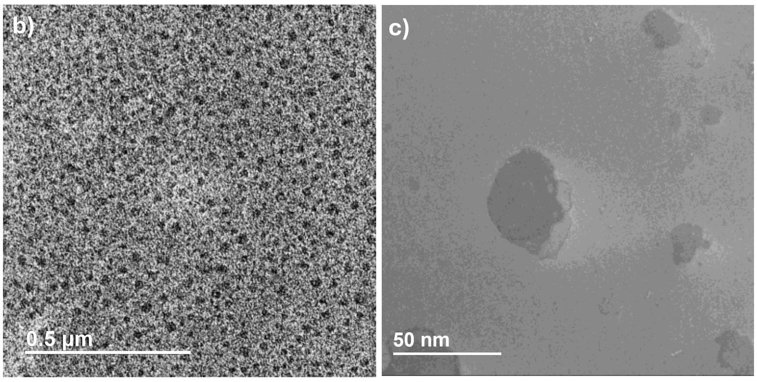

Figure S2. (**a**) AFM, (**b**) TEM and (**c**) SEM images of **(*S*)-1** in EtOH-H_2O (10/90) mixtures. [**(*S*)-1**] = 1.0.10^{-5} M.

To gain a comprehensive understanding of the polymerization process, UV-Vis, fluorescence, and CD experiments were conducted in mixtures of EtOH- H_2O at varying compositions (Figure S3). In EtOH-H_2O 10/90 (Figures S3a–c), the CD spectrum exhibits a weak Cotton effect (**Agg 0**) (Figure S3a). As the EtOH content increased (up to EtOH: 45%), no significant changes were observed in any of the techniques. However, beyond this composition, clear signs of aggregation emerged, including a strong CD signal with a maxima at 560 nm (**Agg I**) and 475 nm (**Agg II**) (Figure S3b). As the EtOH fraction continued to increase, the intensity of these minima gradually decreased, disappearing completely in samples dissolved in pure EtOH (monomer state) (Figure S3c).

The absorption spectra (Figures S3d–f) followed a similar trend to the CD data. An initial broad absorption band was observed (**Agg 0**) (Figure S3d), which decreased in intensity in the EtOH-H_2O 50/50 mixture (**Agg I**) (Figure S3e). With further increase in EtOH content, the absorption gradually recovered (**Agg II** to monomer state**)**, reaching a sharp and well-defined peak in pure EtOH (Figure S3f).

In the fluorescence spectra (Figures S3g–i), increasing EtOH content led to enhanced emission and a slight hypsochromic shift from 620 nm to 610 nm (**Agg 0**) (Figure S3g). In the EtOH-H_2O 55/45 mixture (Figure S3h), a marked increase in fluorescence intensity was observed, attributed to the transition from **Agg I** to **Agg II**. Further increase in EtOH content above 70% resulted in a milder enhancement of emission (monomer state) (Figure S3i).

Overall, these experiments reveal the presence of two distinct processes occurring at around EtOH-H_2O 50/50 composition. The first is characterized by the pronounced emergence of a minimum at 560 nm in the CD spectrum, attributed to the formation of **Agg I**. The second process is marked by the appearance of a new minimum at 475 nm, suggesting the formation of a different aggregate, **Agg II**.

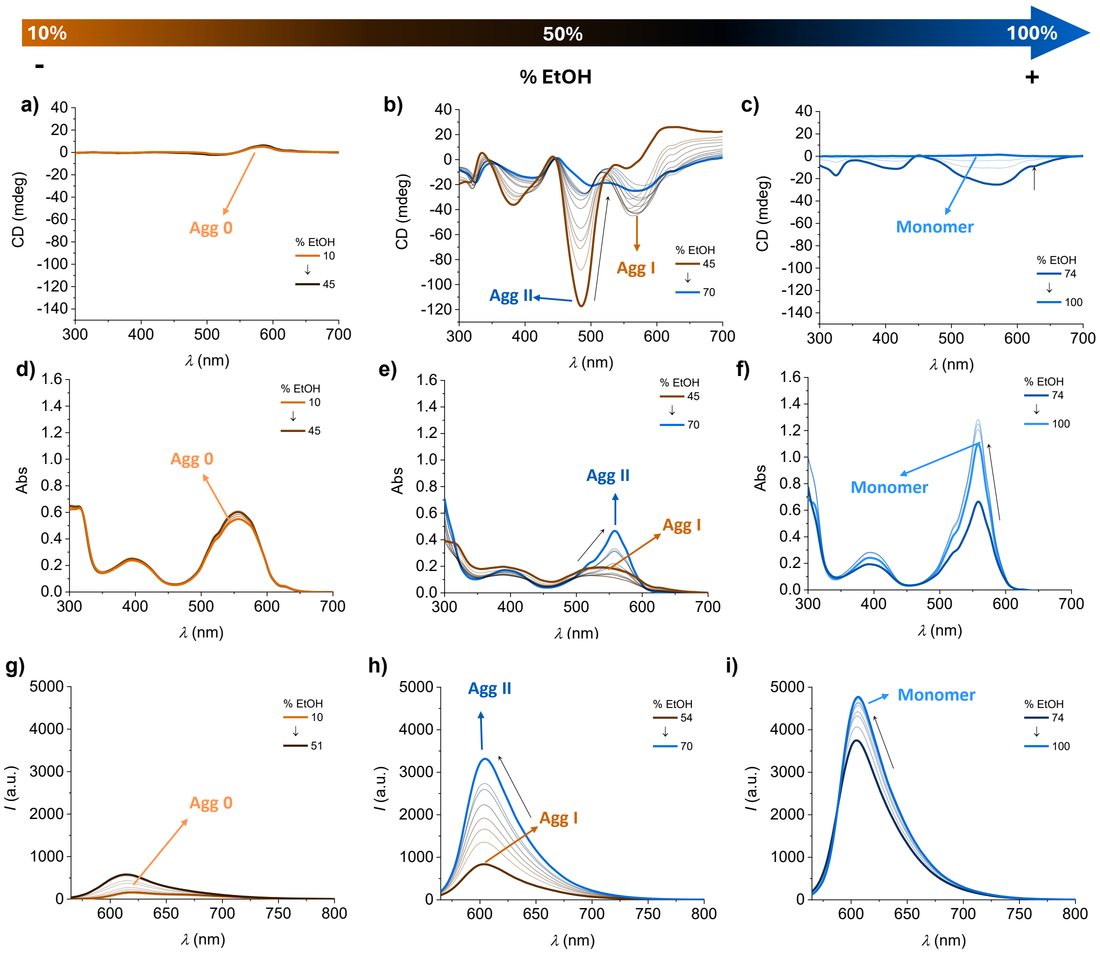

Figure S3. Solvent dependent experiments of **(*S*)-1** in different mixtures of EtOH and H_2O measured by (**a,b,c**) CD (**d, e, f**) UV Vis and (**g, h, i**) fluorescence spectroscopies (λ_{ex} = 550 nm). Left panels (**a,d,g**) correspond to EtOH/H_2O mixtures from 10/90 (starting conditions) to 45/55 composition. Middle panels (**b,e,h**) correspond to EtOH/H_2O mixtures ranging from 45/55 to 70/30 ratios. Right panels (**c,f,i**) correspond to EtOH/H_2O mixtures varying from 74/26 to 100/0 volume fractions. In all cases, [**(*S*)-1**] = $1.0\cdot10^{-5}$ M, *T* = 20 ºC, and l = 1 cm.

1.2 Time-dependent experiments

To analyze the kinetic effect of the EtOH-H_2O mixture 55/45, the solution was prepared *in situ* by direct mixing and spectra were recorded every minute at 20 ºC (Figure S4). Initially, a minimum at 560 nm (**Agg I**) appeared in the CD spectrum (Figure S4a), which stabilized after 30 minutes, as can be seen in the plotted trend (Figure S4c). Subsequently, this minimum began to decrease as the minimum at 475 nm grew (**Agg II**), which stabilized after 60 minutes.

Upon removing the cuvette, a visible precipitate was observed, suggesting the formation of large aggregates in this second process. In contrast to CD results, the absorption measurements (Figure S4b,e), characterized by the decrease in intensity and a blue shift from the monomeric pattern, did not enable to distinguish the two processes. The corresponding time-dependent trend (Figure S4d) highlights a transition that aligns with the first process observed by CD spectroscopy.

In fluorescence spectroscopy (Figure S4c,f) the initial emission band centred at l = 615 nm experiences a hypsochromic shift to 605 nm and a rise in intensity corresponding to the formation of **Agg I**. This signal afterwards diminishes in intensity due to the formation of **Agg II**, leading to the formation of insoluble species around t = 100 min, as can be seen by the irregular trend in emission shown at that time in Figure 4f.

Time-resolved linear dichroism (LD) experiments were also conducted to evaluate the potential formation of large, anisotropic or chiral aggregates associated with the observed precipitation. However, no significant LD signal was detected within the two-hour measurement window.

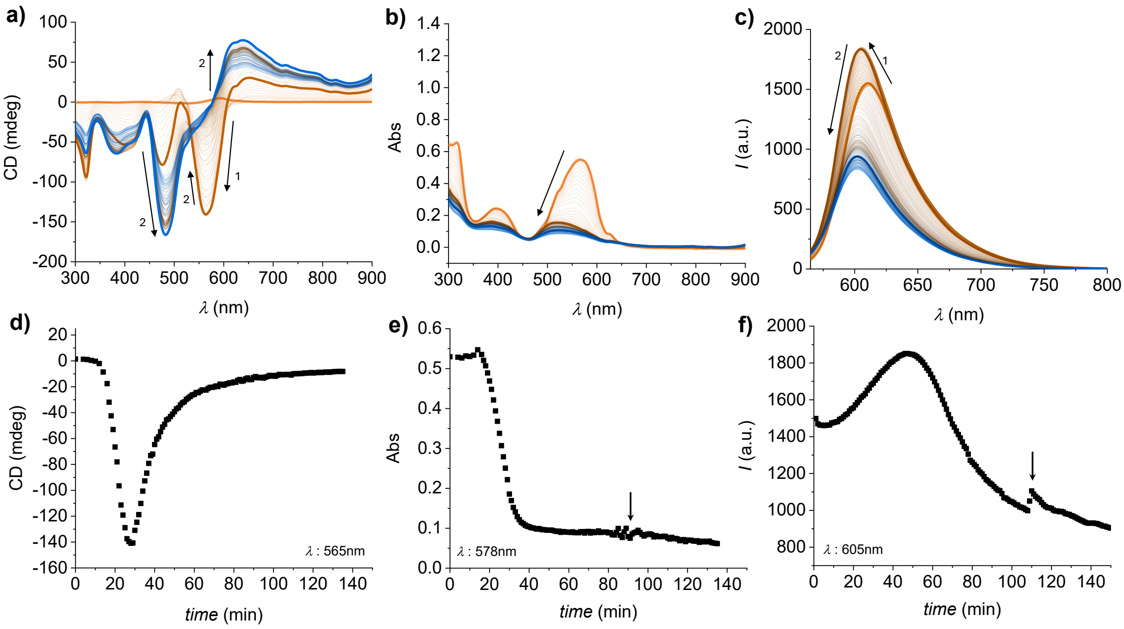

Figure S4. Time-dependent experiment in (**a**) CD, (**b**) UV Vis and (**c**) fluorescence spectroscopies (□$_{ex}$ = 550 nm) of **(*S*)-1** in EtOH-H_2O (55/45) mixture. (**d**) (**e**) and (**f**) denote the changes with time at selected wavelengths in CD, UV Vis and fluorescence spectroscopies, respectively. [**(*S*)-1**] = 1.0·10^{-5} M, (20 ºC, l = 1 cm for all experiments). The arrows in **e** and **f** indicate precipitation of the sample.

To further characterize **Agg I** and **Agg II** and assess their evolution with time, a variety of microscopy techniques (TEM, AFM and SEM) were employed. The samples analysed by the three techniques were dissolved in the EtOH-H_2O 55/45 and deposited at different times, permitting to apply a consistent comparison of their morphological evolution.

Samples prepared **20 minutes** after mixing exhibited longitudinal structures with a diameter of 500 nm, approximately in all the techniques (Figure S5). Furthermore, these aggregates exhibited helical twists, as clearly seen in TEM and SEM images. We attribute this morphology to the spectroscopic features attributed to **Agg I.**

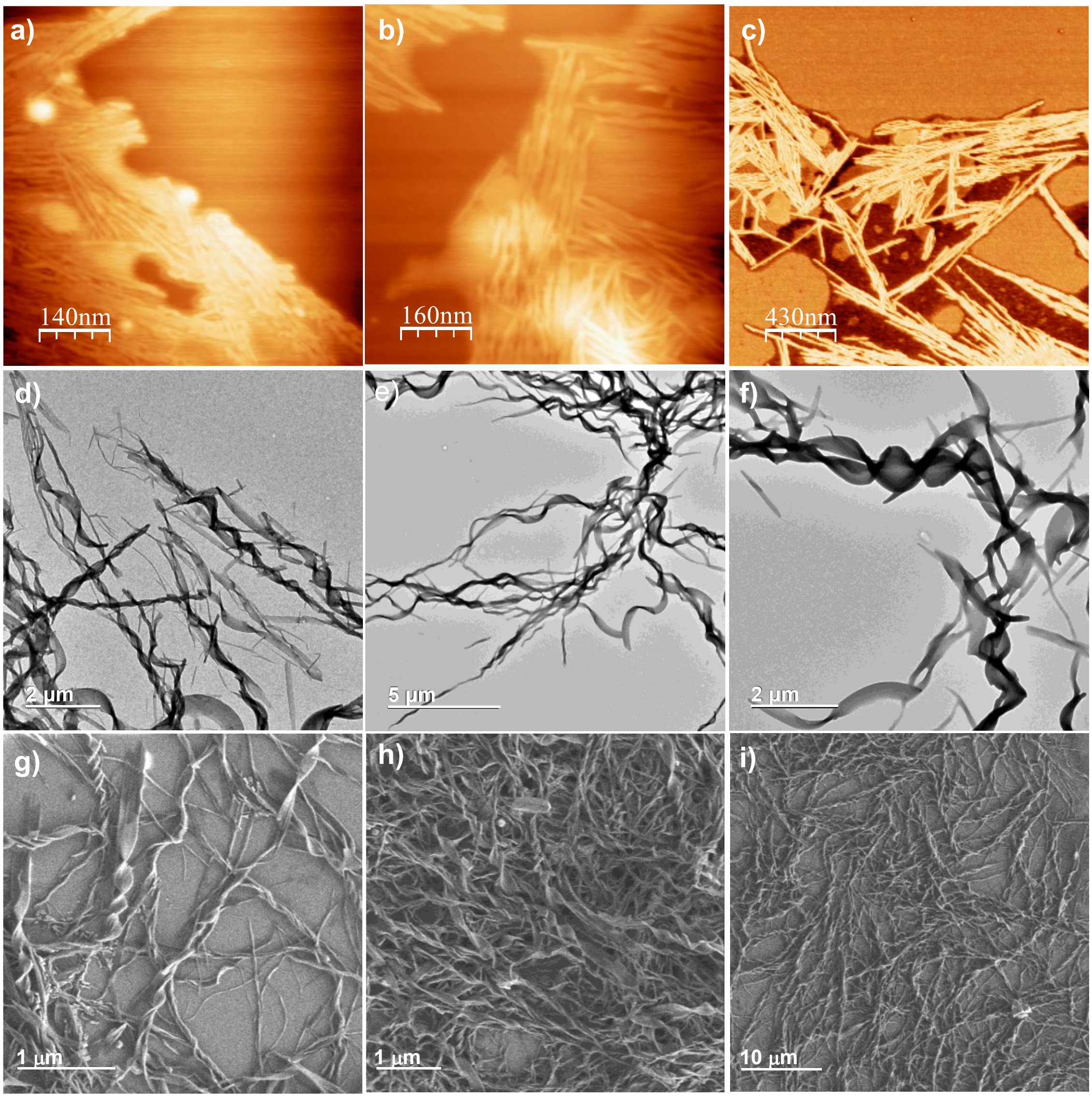

Figure S5. (**a, b, c**) AFM, (**d, e, f**) TEM and (**g, h, i**) SEM images of the aggregates (**Agg I**) formed by **(*S*)-1** in EtOH/H_2O 55/45 mixtures at $1.0 \cdot 10^{-5}$ M after **20 mins** of the mixing equal volumes of the EtOH and EtOH/H_2O 10/90 mixtures

When the samples were deposited **60 mins** after the solution preparation, the helical aggregates are still discernible. However, some pseudo crystalline 2D structures start to emerge (Figure S6). We attribute these morphologies to the spectroscopic features attributed to **Agg I** in the case of the helical fibres and to **Agg II** for the 2D structures**.**

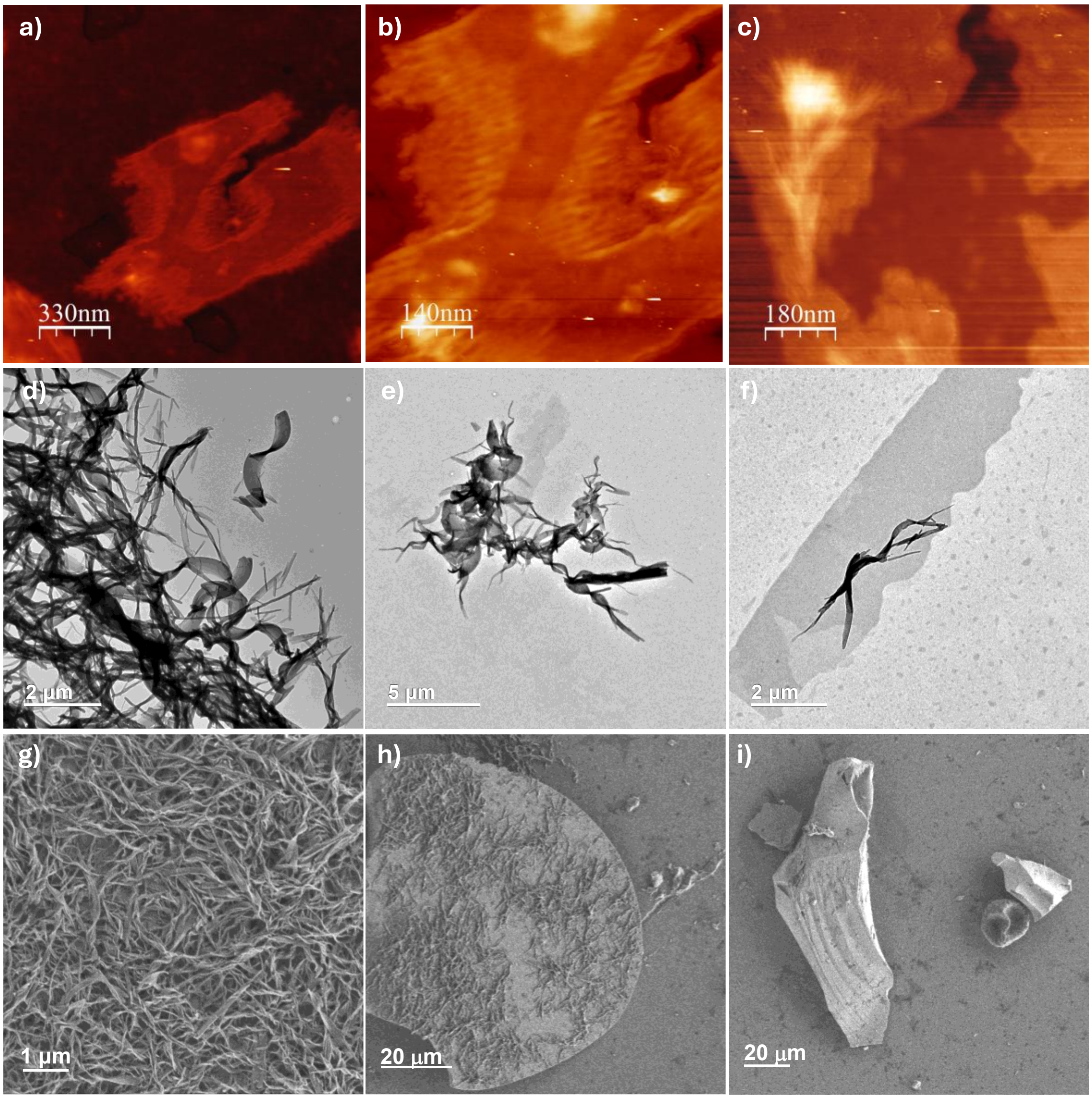

Figure S6. (**a, b, c**) AFM, (**d, e, f**) TEM and (**g, h, i**) SEM images of the aggregates (**Agg I** and **Agg II**) formed by **(*S*)-1** in EtOH/H_2O 55/45 mixtures at $1.0 \cdot 10^{-5}$ M after **60 mins** of the mixing equal volumes of the EtOH and EtOH/H_2O 10/90 mixtures

When the samples were deposited **120 mins** after the solution preparation, the helical aggregates have disappeared, being only noticeable the presence of 2D lamellar structures in all the microscopic techniques (Figure S7). This morphology is consistent with the formation of **Agg II** as shown by the different spectroscopic techniques**.**

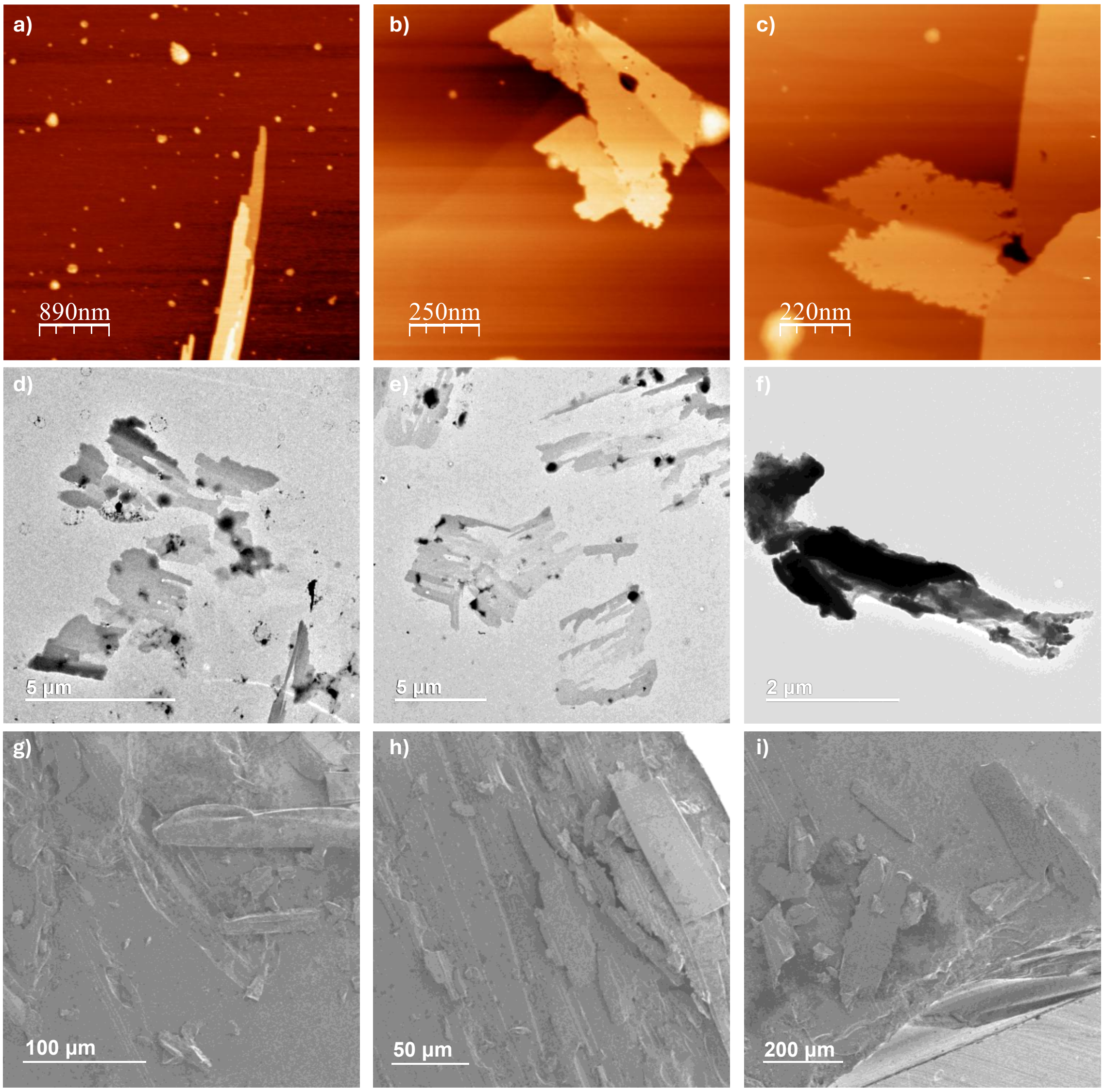

Figure S7. (**a, b, c**) AFM, (**d, e, f**) TEM and (**g, h, i**) SEM images of the aggregates (**Agg II**) formed by **(*S*)-1** in EtOH/H_2O 55/45 mixtures at $1.0 \cdot 10^{-5}$ M after **120 mins** of the mixing equal volumes of the EtOH and EtOH/H_2O 10/90 mixtures.

We afterwards assessed the effect of preparation protocol, concentration, temperature and application of different cooling rates and time evolution in the self-assembly pattern of **(*S*)-1** in the EtOH-H_2O 55/45 mixture.

With the aim to start from a monomeric state, we increased the initial concentration of the monomeric solution of **(*S*)-1** in the EtOH and we mixed it directly with H_2O to yield the $1.0.10^{-5}$ M EtOH-H_2O 55/45 mixture (Figure S8). The formation of **Agg I** and **Agg II** was observed in an analogous manner to that of the previous protocol, proving that this phenomenon accounts from the concrete conditions employed and not the presence of the preformed aggregate **Agg 0**.

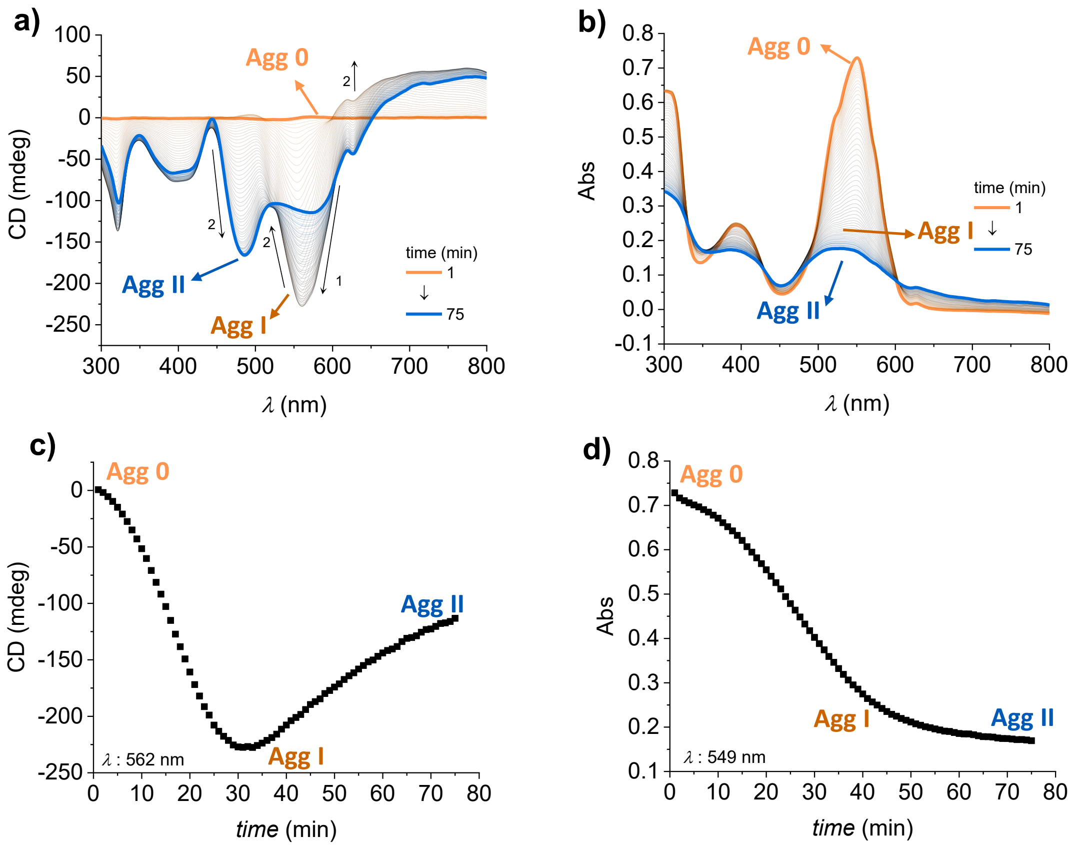

Figure S8. Kinetics at 20 ºC followed by (**a**) CD and (**b**) UV Vis spectroscopies after direct mixing a $2.0\cdot 10^{-5}$ M monomeric solution of **(*S*)-1** in pure EtOH with H_2O to yield a $1.0\cdot 10^{-5}$ M EtOH/H_2O 55/45 composition (l = 1 cm). (**c**) and (**d**) denote the changes with time at selected wavelengths in CD and UV Vis spectroscopies, respectively.

We afterwards explored the role of concentration and temperature in order to gain insight into the type of self-assembly pathway experienced by **(*S*)-1**. A $5.0\cdot 10^{-6}$ M EtOH-H_2O 55/45 mixture (2 x concentration diluted) was prepared in an analogous manner than the previous protocol, being only discerned the formation of **Agg I** (Figure S9). We consider that this behavior could be symptomatic of a hierarchical self-assembly process, although the partial precipitation of the sample cannot be neglected.

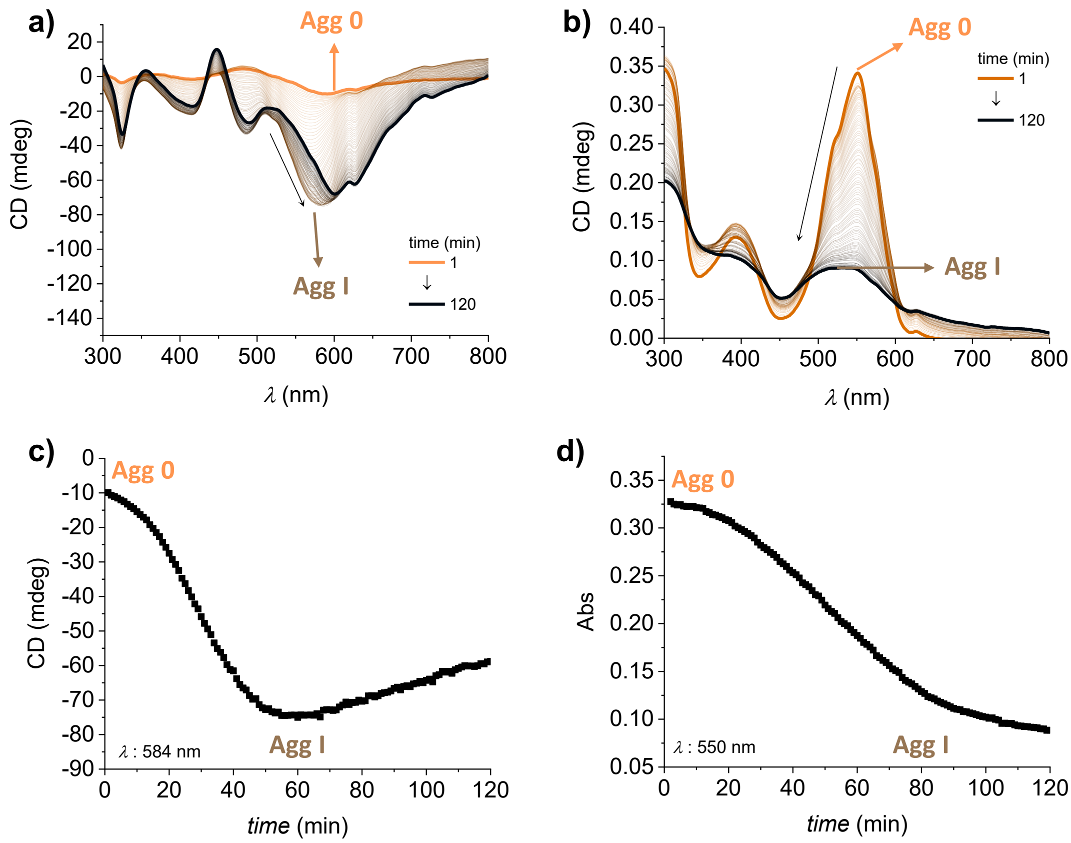

Figure S9. Kinetics at 20 ºC followed by (**a**) CD and (**b**) UV Vis spectroscopies after direct mixing a $1.0 \cdot 10^{-5}$ M monomeric solution of **(*S*)-1** in pure EtOH with H_2O to yield a $5.0 \cdot 10^{-6}$ M EtOH/H_2O 55/45 composition (l = 1 cm). (**c**) and (**d**) denote the changes with time at selected wavelengths in CD and UV Vis spectroscopies, respectively.

We subsequently looked into the effect of temperature after the direct mixing of equal volumes of a $1.0 \cdot 10^{-5}$ solution of **(*S*)-1** in EtOH and a $1.0 \cdot 10^{-5}$ solution of **(*S*)-1** in EtOH-H_2O 10/90 mixture to afford the $1.0 \cdot 10^{-5}$ solution of **(*S*)-1** in EtOH-H_2O 55/45 mixture. Performing this mixing at 30 ºC yields **Agg II** directly, with no detection of **Agg I** at these scale times, whereas in the 40 ºC kinetics **Agg I** is initially observed and then evolves to **Agg II**, being this process slower than at 20 ºC (Figure S10).

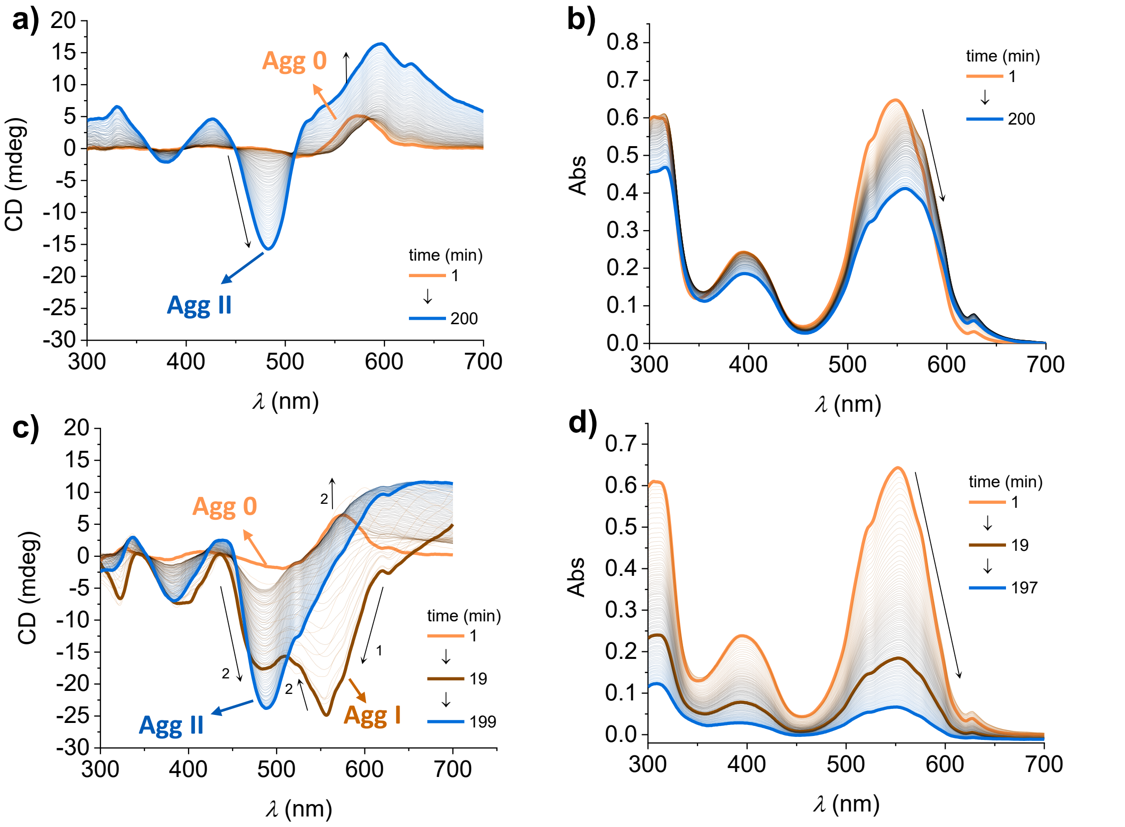

Figure S10. Kinetics at (**a**, **b**) 30 ºC and (**c**, **d**) 40 ºC followed by (**a**, **c**) CD and (**b**, **d**) UV-Vis spectroscopy after direct mixing to yield the $1 \cdot 10^{-5}$ M EtOH/H_2O 55/45 composition (l = 1 cm).

Finally, we also evaluated the formation of these aggregates after going to the monomeric state and applying distinct cooling rates (1 K min^{-1} and 5 K min^{-1}). After direct mixing the solutions, these were subjected to heating to 70 ºC to lead to the depolymerization of the sample. The application of different cooling rates always yielded a lower dichroic signal accompanied by substantial decrease of the UV absorption, that is a result of the formation of aggregates films in the glass of the cuvette. Still, the moderate chiroptical signals observed seem to correspond those of **Agg II**. Following the evolution of this signal with time seems to further corroborate this trend.

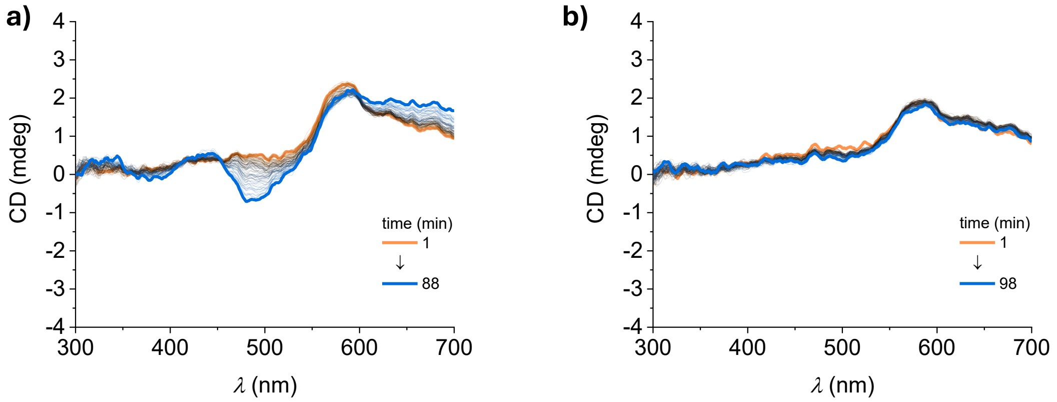

Figure S11. Kinetics at (**a**) 10 ºC and (**b**) 40 ºC followed by CD spectroscopy after applying a 1 K min^{-1} cooling rate to the target temperature in the $1 \cdot 10^{-5}$ M EtOH/H_2O 55/45 composition.

2. Solid state studies

2.1 Polarized Optical Microscopy (POM)

POM experiments were initially performed to analyse the crystallization of the material as a function of temperature.

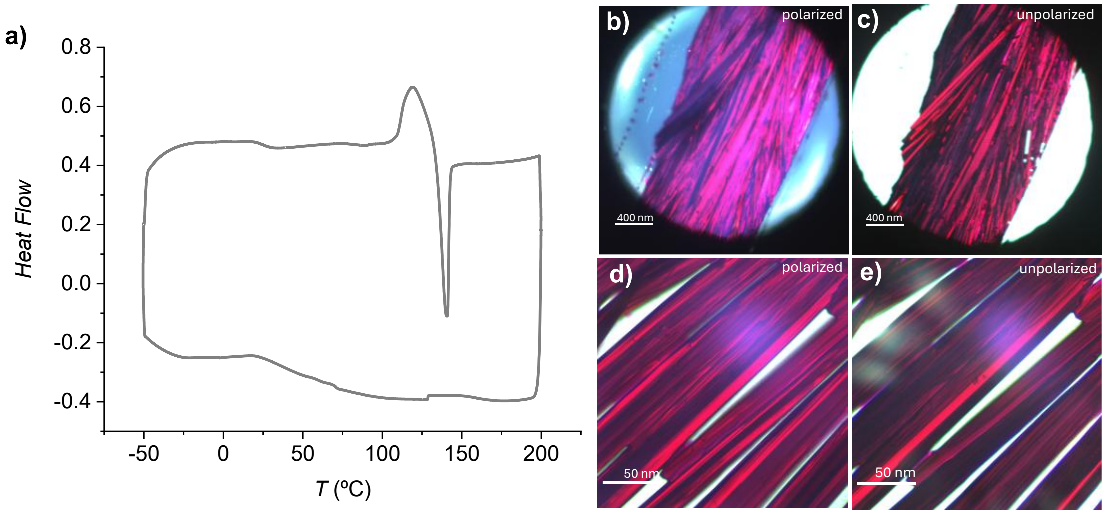

Figure S12 (**a**) DSC spectrum of **(*S*)-1**, unraveling its crystallization at 140 ºC. (**b**-**e**) POM images of the crystals formed by **(*S*)-1** obtained after the heating/cooling cycles.

2.2 Powder XRD experiments

Distances were calculated using Bragg's law for fist-order reflections (n = 1):

Using Bragg's law for first-order reflections (n = 1):

d = λ / 2·sen ϴ

where λ = 1.5406 Å because the instrument use Cu Kα radiation.

Table S1. Distances calculated for the main diffraction peaks of the diffractogram obtained for single crystals grown in acetonitrile/ethanol.

**ϴ angle**	**Distances (Å)**
19.29 º	4.60
20.76 º	4.28
21.45 º	4.14
28.00 º	3.54
32.57 º	3.18

Table S2. Distances calculated for the main diffraction peaks of the diffractogram obtained for aggregates prepared in ethanol.

**ϴ angle**	**Distances (Å)**
6.29 º	14.05
7.80 º	11.33
11.12 º	7.95
12.41 º	7.13
18.10 º	4.90
18.61 º	4.76

Table S3. Distances calculated for the main diffraction peaks of the diffractogram obtained for aggregates prepared in ethanol/H_2O (90/10).

**ϴ angle**	**Distances (Å)**
6.35 º	13.91 (very intense)
7.92 º	11.16 (very intense)
8.78 º	10.07 (lower intensity)
18.16 º	4.88 (very weak)
18.70 º	4.74 (very weak)
21.98 º	4.04 (very weak)
25.82 º	3.45 (very weak)

Table S4. Distances calculated for the main diffraction peaks of the diffractogram obtained for aggregates prepared in ethanol/H_2O (10/90).

**ϴ angle**	**Distances (Å)**
6.20 º	14.23 (very intense)
7.69 º	11.48 (very intense)
8.63 º	10.23 (very intense)
9.53 º	9.27 (very intense)
17.96 º	4.93 (very weak)
18.47 º	4.80 (very weak)
21.25 º	4.18 (very weak)
21.77 º	4.08 (very weak)

Table S5. Distances calculated for the main diffraction peaks of the diffractogram obtained for aggregates prepared in ethanol/H_2O (55/45) (Agg II).

**ϴ angle**	**Distances (Å)**
6.27 º	14.09 (very intense)
7.73 º	11.43 (very intense)
11.06 º	7.99 (very weak)
12.39 º	7.14 (very weak)
12.88 º	6.87 (very weak)
17.20 º	5.15 (very weak)
18.02 º	4.92 (very weak)
18.57 º	4.77 (very weak)
21.87 º	4.06 (very weak)
25.68 º	3.47 (very weak)
28.57 º	3.12 (very weak)
32.49 º	2.75 (very weak)

2.3 Single crystal XRD experiments

Crystallization conditions: Crystals of **(*S*)-1** were grown using the slow diffusion method. A small vial with 1 mL of **(*S*)-1** in acetonitrile (1 mg/mL) was introduced into a larger vial containing ethanol as precipitant. The system was closed to allow vapor diffusion until equilibration. Single crystals were obtained within a period of 1-2 weeks.

a)

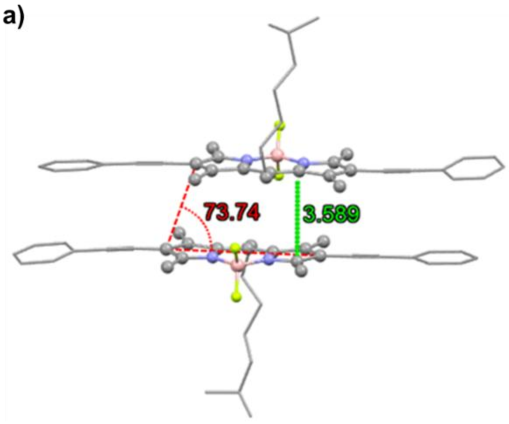

Figure S13. (**a**)View of the dimer formed by **(*S*)-1**, arranged in an H-type aggregate as indicated by a slip angle of 73.7°.

2.3 Spectroscopic features of crystals

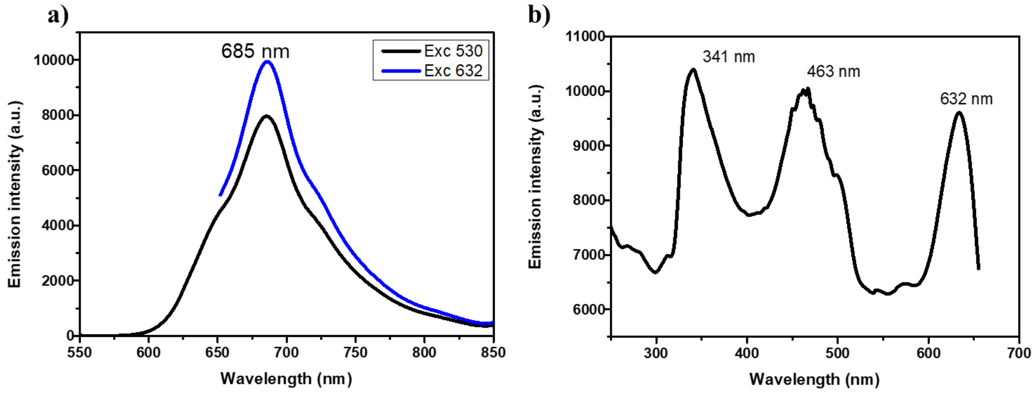

Figure S14. (**a**) Emission and (**b**) excitation spectra of ***(S)*-1** crystals. Emission spectra were recorded at different excitation wavelengths (530 and 632 nm). Excitation spectrum was recorded at emission wavelength of 632 nm.

2.4 Optical waveguiding properties

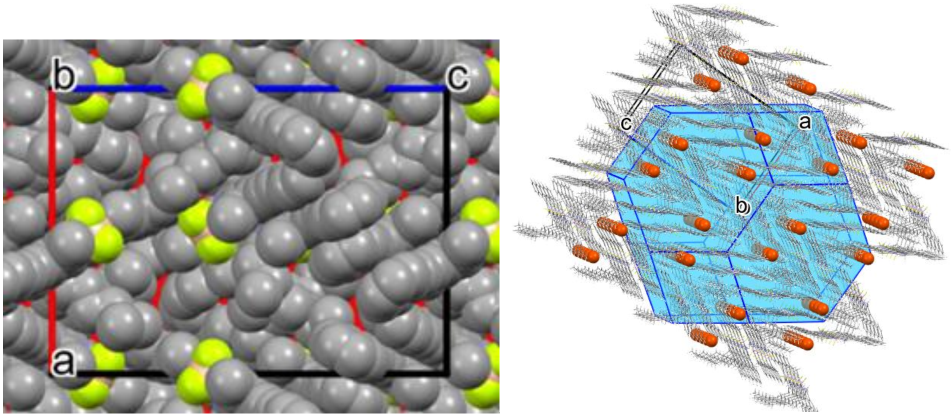

Figure S15. (**a**)Fill representation of the structures showing microchannels (red color) along b-axis direction (**b**) Representation of microchannels depicted with red balls

2.5 Crystallographic information of (*S*)-1 structure

Table S6. Structural parameters for the non-covalent interactions in **(*S*)-1** derivative. Ct = centroid. β is the angle formed by H-centroid and H-plane lines in the case of the CH···π interaction and the angle formed by the centroid-centroid and centroid-plane lines in the case of the π-π stacking.

**CH···F Interactions**			
**DH···A**	**d (H···A) Å**	**d (D···A) Å**	**a (DHA)º**
C36-H36A···F1	3.00	3.86	146.3
C28-H28···F1	3.24	3.56	102.9
C5-H5A···F1A	2.68	3.58	157.4
C13-H13···F1A	2.77	3.31	118.4
C14-H14···F1A	2.70	3.27	120.4
C30-H30A···F1A	2.85	3.28	107.4
C6A-H6AB···F1A	2.68	3.13	109.4
C14A-H14A···F2A	3.13	3.51	106.8
C20A-H20E···F2	2.62	3.55	161.0
C21A-H21F···F1A	2.45	3.08	123.2
C21A-H21F···F1A	2.54	3.15	121.8
C28A-H28A···F2	2.77	3.37	123.1
C30A-H30D···F2	2.55	3.36	140.4
**C-H···π interactions**			
**Groups**	**d (H-Ct) Å**	**d(H-plane) Å**	**bº**
C5-H5B···(C24A-C29A)	3.64	3.36	28.9
C6A-H6A···(C9A-C14A)	3.15	2.97	19.5
C5-H5C···(C25A-C29A)	3.51	3.49	6.1
C12A-H12A···(N1A-C4A)	3.51	3.15	26.2
C13A-H13A···(N1A-C4A)	3.50	3.29	19.9
C20A-H20F···(C9-C14)	3.38	3.31	11.7
C21-H21C··· (C24-C29)	3.16	3.05	15.2
C28-H28··· (N2-C19)	3.43	3.34	13.1
C34-H34B···(N2A-C19A)	3.69	3.34	25.1
C20A-H20D···(C9-C14)	3.46	3.34	15.1
C5A-H5AA···(C7≡C8)	3.24		
C21-H21C···(C22≡C23)	2.99		
C20-H20B···(C22A≡C23A)	3.39		
C27-H27···(C22≡C23)	3.38		
C32-H32A···(C22A≡C23A)	3.36		
C12A-H12A···(C7A≡C8A)	3.45		
C32A-H32F···(C7≡C8)	3.16		
C32A-H32E···(C7≡C8)	3.32		
**π-π stacking**			
**Groups**	**d(Ct-Ct) Å**	**d(Ct-Plane) Å**	**b (a) º**
(N1-C4) ···(N1A-C4A)	4.00	3.61	25.2
(N2-CA9)···(N2A-CA9A)	4.00	3.59	26.1
(C9-C14) ···(C9A…C14A)	4.07	3.28	36.3
(C24-C29) ···(C24A…C29A)	4.01	3.77	19.9

Table S7. Crystal data and structure refinement for **(*S*)-1**.

Empirical formula	$C_{38}H_{41}BF_2N_2$
Formula weight	574.54
Temperature/K	296.15
Crystal system	orthorhombic
Space group	$P2_12_12_1$
a/Å	20.637(5)
b/Å	11.267(3)
c/Å	28.605(6)
α/°	90
β/°	90
γ/°	90
Volume/Å^3	6651(3)
Z	8
ρ_{calc}g/cm^3	1.147
μ/mm^{-1}	0.074
F(000)	2448.0
Index ranges	-22 ≤ h ≤ 24, -13 ≤ k ≤ 13, -34 ≤ l ≤ 30
Reflections collected	31106
Independent reflections	11641 [R_{int} = 0.1360, R_{sigma} = 0.2101]
Data/restraints/parameters	11641/732/777
Goodness-of-fit on F^2	0.920
Final R indexes [I≥2σ (I)]	R_1 = 0.0841, wR_2 = 0.1800
Largest diff. peak/hole / e Å^{-3}	0.21/-0.21

3. References

1. B.-A. Saint v8.37, APEX3 v2016.1.0. Madison, Wisconsin, USA.
2. L. Krause, R. Herbst-Irmer, G. M. Sheldrick and D. Stalke, *J. Appl. Crystallogr.*, 2015, **48**, 3-10.
3. O. V. Dolomanov, L. J. Bourhis, R. J. Gildea, J. A. K. Howard and H. Puschmann, *J. Appl. Crystallogr.*, 2009, **42**, 339-341.
4. Mercury 2025.1.0 of Cambridge Crystallographic Data Centre
5. A. T. Rösch, Q. Zhu, J. Robben, F. Tassinari, S. C. J. Meskers, R. Naaman, A. R. A. Palmans and E. W. Meijer, *Chem. Eur. J.*, 2021, **27**, 298-306.
